# Seafood consumption changes and COVID-19 impact index in West Nusa Tenggara, Indonesia

**DOI:** 10.1371/journal.pone.0280134

**Published:** 2023-01-18

**Authors:** Stefan Partelow, Ben Nagel, Adiska Octa Paramita, Nurliah Buhari

**Affiliations:** 1 Leibniz Centre for Tropical Marine Research (ZMT), Bremen, Germany; 2 Jacobs University, Bremen, Germany; 3 University of Mataram, Lombok, West Nusa Tenggara, Indonesia; International Maize and Wheat Improvement Centre: Centro Internacional de Mejoramiento de Maiz y Trigo, MEXICO

## Abstract

This empirical study examines seafood consumption patterns in the province of West Nusa Tenggara, Indonesia at the regency level, and analyzes changes in consumption patterns during the COVID-19 (SARS-CoV-2) virus outbreak. We used a stratified semi-random general population survey administered online through mobile devices from November 24th–December 31st 2020 for rapid assessment and dissemination, which received 1518 respondents. Our findings enabled us to generate a COVID-19 impact index at the regency level, indicating an urban-to-rural gradient in the degree of change in seafood consumption patterns, with rural areas incurring more changes. During COVID-19, 61% of respondents ate less seafood than normal, 66% stated seafood was more expensive, and 37% stated that the seafood they normally buy was not available. Respondents also bought 5% less fresh or raw seafood, and 4.3% more pre-cooked seafood products during the pandemic. Traditional markets, mobile vendors, and food stands remain the most frequent access points for seafood, although access decreased during the pandemic for all, with mini- and supermarket access slightly increasing. Raw and fresh seafood purchases from travelling merchants decreased 12.5% during the pandemic. A larger percentage of women (~10% more than men) eat fish at least once per week, and women eat a larger diversity of seafood products. However, men classified themselves on average in a higher income class than women both before and during the pandemic, and men were significantly more likely to agree that they had enough money to buy the food they wanted during the pandemic. Overall, respondents who indicated eating a higher frequency of fish per week, were significantly more likely to agree that they ate less fish during the pandemic. Respondents on Sumbawa island were significantly more likely to agree that the fisheries products were not available during the pandemic.

## 1. Introduction

Fisheries in Indonesia support millions of livelihoods, and seafood products are estimated to be the largest source of food protein at 14% [1], suggesting that changes in access to seafood products may affect dietary compositions, food security and overall nutrition. Estimates of fish consumption per capita per year in Indonesia range from 20.7 kg [2] to 43.88kg [3] to 44.17kg [4], with an average growth rate of consumption at 6.68% from 2010–2019 [5]. The national daily average of protein consumption from 2014–2020 was 59 grams, of which 7.9 grams come from fish, although these averages vary substantially by province and urban-rural gradients [6]. Nonetheless, with over 270 million people, Indonesia is ranked second highest in total seafood consumption per year after China. Indonesia is the second largest producer of capture fisheries tonnage after China, and the third largest aquaculture producer after China and India [7, 8]. However, aquaculture and capture fisheries trends vary widely across provinces. For example, over 80% of tonnage comes from capture fisheries in West Papua, Papua and North Sumatera, and over 80% comes from aquaculture in West Nusa Tenggara, North Kalimantan and South Sulawesi [3]. Less is known about seafood consumption trends and value chains beyond production.

Despite high production, data on seafood consumption patterns such as what species are purchased, where (i.e., market types, restaurants) and in what form (i.e., fresh, dried, canned) is scarce beyond local studies. Similarly, little if any published literature on the perceived value of, or preferences for, locally produced seafood consumption exists. In urban areas, larger supermarkets offer diverse non-local food options, often changing dietary compositions, while in low and middle income (often rural) areas, traditional markets and local restaurants (*warung*) are still dominant [9]. Urban households in Indonesia also tend to consume more animal-based protein sources as compared to rural households which are more reliant on plant-based protein [10]. Early in the pandemic, the World Food Program published a report on Indonesia’s COVID-19 impacts [1] stating that during the earlier 2020 pandemic lockdown, “most surveyed households were still able to access rice and other staple foods (97%) […]; meat, fish, and eggs (88%); and fruit and vegetables (98%) […] However, 31% of households experienced ‘some shortage of food’ and 38% admitted to ‘eating less than they should over the previous week due to lack of money’. Poorer households reported a higher prevalence and severity of food insecurity, along with households experiencing loss of income.” The report further states that fish prices fell in markets due to reduced demand in restaurants and hotels, disruptions to cold-storage supply chains and distribution networks beyond production areas, whereas locally (in production areas) fish supply often increased for the same reasons. Furthermore, a recent study by Syafiq et al., [11], surveying 517 people in Jarakta and Depok, indicated that 65% of respondents had some level of food insecurity during the COVID-19 pandemic, mostly related to income declines reducing food purchasing power. This is supported by a literature review [12] of Indonesian food security impacts from COVID-19 and the effect of government policy strategies, indicating that decreased purchasing power impacts the food security of vulnerable groups the most, and that further policy actions should focus on strengthening the financial resilience of local smallholder food systems.

The first case of COVID-19 was reported in Indonesia on March 3rd, 2020, spreading rapidly across 34 provinces through April 9th, leading to a declaration of a state national disaster and health emergency [13]. However, Indonesia’s response at the beginning did not include strict containment measurements [14]. As of March 2021, the number of confirmed cases in Indonesia reached nearly 1,5 million, with more than 40 thousand deaths (www.covid19.go.id). Indonesia has never applied a national lockdown, instead large scale social restrictions (*Pembatasan Sosial Berskala Besar*) have been imposed in several regions across Indonesia with different policies and protocols [13]. Nonetheless, Indonesia’s economy has been severely affected by the COVID-19 pandemic due to large-scale mobility restrictions, creating the largest economic catastrophe since the Asian financial crisis two decades ago [13].

This study examines the seafood consumption patterns in West Nusa Tenggara province at the regency level, and the changes incurred due to the social and economic changes resulting from the COVID-19 (SARS-CoV-2) virus outbreak. We conduct a general population level survey administered online through mobile devices using Whatsapp and Facebook. Indonesia has 34 provinces (provinsi), each containing regencies (kabupaten) and cities (kota) representing the second-level administrative division, each subdivided into third-level districts (kecamatan). The data for this study was collected between November 25th and December 31st, 2020 (S1 Fig in S1 File) in West Nusa Tenggara province, including eight districts and two cities. Mataram city is considered a high-risk COVID-19 area compared to other Indonesian cities, while North Lombok and West Sumbawa are medium risk, and the rest are low-risk areas (www.covid19.go.id). West Nusa Tenggara is home for over 5.3 million people and known for its growing tourism and aquaculture economies. It includes the two main islands of Lombok and Sumbawa with a combined 8 regencies and 2 cities. Comparing across provinces, West Nusa Tenggara has highly productive fisheries [15]. The province also has the second highest consumption of fish per capita per year in Indonesia with 12.83% average annual growth rate from 2010–2019 [5]. As of 2015, the province was ranked 6th out of 34 in total aquaculture production, and 5th in its aquaculture production growth rate at 29.63% [3]. Out of all fisheries production in the province, ~84% is from aquaculture when seaweed culture is included.

The province is diverse in its economic development, ranging from Mataram (Lombok), a city of ~500,000 people with diverse economic opportunities, established tourism areas and a large university, to rural agriculture, fishing villages and remote forests throughout much of Lombok and Sumbawa. According to official data collected in March 2020, the daily consumption for fresh and processed seafood in West Nusa Tenggara was 9.99 grams of protein per day, along with beef (0.07g), chicken (2.04g), and soya based protein (4.31g) [6]. Protein in the region is primarily purchased fresh and cooked at home (~71%), with higher percentage of home cooked food in rural areas (~75%) [6]. However, how and where seafood is accessed, as well as granular data on species consumed in different forms has, to our knowledge, yet to be examined at the regency or district level. The research in this survey aims to answer two primary questions:

1. What are the seafood consumption patterns in the province of West Nusa Tenggara, Indonesia at the regency level?
2. Has COVID-19 changed seafood consumption patterns?

### 1.1 COVID-19 impacts on seafood availability and consumer behavior

Food security and access during COVID-19 is a global issue with varying severity across regions [16–20]. The impacts on the seafood sector are already being documented with diverse outcomes [21–26]. Mobility and economic interconnectivity across borders is essential for linking fishing crews, boats, ports, markets and consumers, which has almost entirely been stagnated [23, 27]. In many cases, complete fisheries closures due to health and safety concerns, market restrictions or supply chain interruptions have created limited access to seafood products and job losses [24, 28–30]. Supply and value chain vulnerability have stagnated seafood distribution and access. In study on the lessons learned across seven small-scale fisheries supply chains during COVID, Bassett et al., [31] argue that there is a “need to support development and maintenance of a diversity of distribution channels during ‘normal’ times to meet the need for resilient local distribution systems during crises,” (p. 7). In other cases, new institutions have emerged as adaptive strategies such as local seafood networks, direct sales, switching species and food sharing [32, 33]. Negative coping strategies have also been adopted, particularly for small-scale producers such as “selling productive assets, borrowing money, or reducing health, education or food expenses/ consumption,” ([34], p. 809). The first global review of food security impacts and impact pathways by Béné et al., [20] provides a four stage series of possible event pathways and eventual final impacts facing food system actors. The final consumer impacts are: reduced self-efficacy, domestic violence, degradation in food choice and diversity, reduction in convenience, increased risk of unsafe food or a forced shift to more expensive outlets. Overall, the study concludes that the resilience of food systems was achieved, but at great costs to the majority of the actors who cope with severe activity disruptions and some subsequent financial losses [20].

Early pandemic findings indicated that COVID-19 has shifted how seafood is being accessed, what type and how much [20, 23, 24, 31]. In Indonesia, fisheries are thought to be one of the most severely affected sectors after agriculture and forestry [13], due to declines in fish prices across provinces (up to 50%) and declines in export and local business (hotels and restaurants) demand [15]. On the supply side, the impact of pandemic has caused delays in delivery of fresh seafood products, as well as decreased number of fishing days, volume of catches, and income of fishers and aquaculture farmers [35]. Bassett et al., [36] provide findings on how exports of fisheries products in Langkat, North Sumatra were shut down, leading to an oversupply of fish in local markets at substantially reduced prices and income. These findings were further supported by Azhari and Muis [37], who also found that fishers who couldn’t sell or profit from their fish would distribute them to family or salt them for storage. Campbell et al., [38] show that the total catch weight and price per kilogram of fish declined in Southeast Sulawesi, suggesting access to fish was lower than usual and more expensive. Farradia and Sunarno [39] suggest a shift towards purchasing fish online during COVID-19 due to market closures and safety concerns. However, one of the key messages in the World Food Program [1] report on Indonesia is that limited data availability on the prices and availability of nutritious food makes examining access to food economically and physically challenging, especially during COVID-19. More generally, Indonesian consumers are showing a shift towards modern and larger markets and chains, although traditional markets, *warung* (local food stands), but wet markets still dominate food access [40]. Preferences for locally produced and traditional foods are still strong in the country, although varied by province and ethnic group [41]. Fish products are generally viewed as a part of a healthy diet, and because they are kept at affordable prices due to fish diversity and abundance in Indonesia generally, they are consumed regularly [42].

## 2. Methods

We sampled the general population in the province of West Nusa Tenggara and its 8 regencies and 2 cities, which includes the larger islands of Lombok and Sumbawa. The survey aimed to conduct a rapid assessment of seafood consumption patterns before and during the COVID-19 (SARS-CoV-2) outbreak and closures in 2020. Seafood in this context refers to fresh and processed food products from the sea and aquaculture, including fresh, brackish, and salt water. In Bahasa Indonesia, “seafood” is adopted and used to refer to food sources from the sea that exclude aquaculture products. Due to this, we framed seafood as “all fisheries products from fresh, brackish, and salt waters, including seaweed” in the questionnaire.

The survey was designed on Limesurvey (Version 3.25.10) for online distribution and completion, primarily for mobile devices. We used two different online semi-random sampling strategies: (1) Semi-random snowball sampling through the mobile phone application WhatsApp with more than 50 entry points into the general population, meaning we had the contacts of more than 50 people who were first sent the survey via WhatsApp. Respondents were encouraged to forward the survey to their contacts. (2) Targeted sampling using online advertisements on Facebook, stratified by regency and age to compensate for skews in population representativeness in our mobile app response distribution. To incentivize completion of the survey, we offered all respondents 10,000 Indonesian Rupiah (~ 0.67 euro cents) of mobile phone credit, which was sent to each respondent within 2 weeks of completion to the mobile number provided. Submissions from the same IP address (e.g., the same phone to earn more credit) were blocked by Limesurvey software. The survey was conducted between November 24th and December 31st, 2020. The survey asked respondents to consider their consumption behavior before and during COVID-19 social mobility restrictions, which occured in Indonesia from March 31^st^, 2020 through the survey period.

The survey consisted of two parts (1) demographic and socioeconomic data, and (2) seafood consumption behavior (Section 3 in S1 File). Part 1 collected data on age, gender location, household size, occupation, monthly income, education and monthly expenditures. Part 2 collected data on seafood consumption frequency, changes to due to COVID-19, species preferences, product types preferences and market preferences. Minimarkets and supermarkets are modern self-service stores retailers that sell groceries and daily necessities. Minimarkets have sizes around 100 m2 to 999 m2, meanwhile, supermarkets have bigger sizes around 1,000 m2 to 4,999 m2 and offer more varieties of products. Traditional markets are market places provided by the government to support local farmers and fishers to sell their products. It allows consumers to bargain while buying in large quantities. Mobile merchants sell raw and fresh groceries with cart or wagon on foot or by motorcycle. They usually get supplies from traditional markets or local farmers and fishers. Street vendors are sellers of cooked meals that are prepared by order and located in the pedestrians with a semi-permanent tent for customers to dine in. Food stands (*warung*) sell ready to eat home cooking meals in the permanent infrastructure with affordable prices. Additional information ‘Income class’ and ‘Expenditure class’ were calculated from the changes in income and expenditures before and during COVID-19. For each class, respondents were placed into either ‘Same’, ‘Went up’, or ‘Went down’, based on the raw data. The survey took on average about 5 minutes to complete. The full survey is available in the original Bahasa-Indonesia and English in Section 3 in S1 File.

Descriptive statistics were calculated for all data. Skews in the demographic data were calculated based on actual census data on population per regency as well as age, gender and education distribution at the province level [6]. This data was used to calculate the skew in our sample, and weight the survey data for further analysis. Weighting and further analysis was conducted in the statistical interface R [43], using the ‘base’ package for non-weighted descriptive statistical analysis and the package ‘survey’ [44] which is specifically designed for weighting and analyzing weighted survey data. A single weight needs to be attributed to each survey entry based on its proportional skew compared to actual population percentages. A post stratification weight was calculated for each entry based on each demographic skew (age, location, gender, education) by dividing the population percentage by the sample percentage to get a post-stratification weight for each category. The four post-stratification weights for each entry were then averaged to get a single weight score for each survey entry.

To better understand the demographic and socio-economic characteristics of the respondents in relation to how they answered questions on seafood consumption changes during COVID-19, we fit logistic regression models to the data. A model was fitted for each dependent binary variable (i.e., agree/disagree questions), and reduced to the minimum amount of factors necessary. NA responses (i.e., Don’t eat fish) were dropped. Non-significant factors were removed. Logistic regression models were also calculated within the R package ‘survey’ [44] which is specifically designed for weighting and analyzing weighted survey data.

In order to explore how spatial and demographic factors relate to seafood consumption, we ran ANOVA tests to identify significant variation in diversity of seafood consumption with factors such as region, income, and education. To assess changes in seafood consumption due to COVID-19, responses were compared regarding purchasing patterns of cooked seafood, fresh seafood, as well as individual seafood product types before and during the COVID-19 pandemic. Overall proportions of responses were compared before and during COVID-19 using two-proportion z-tests to identify which specific categories of seafood purchasing location and seafood product type were most significantly impacted by the pandemic.

To assess spatial patterns in seafood consumption changes as a result of COVID-19, an index was developed consisting of seven individual indicators derived from the survey questions. Four of these indicators measured the proportion of respondents per regency who reported due to COVID a: (1) decrease in seafood consumption, (2) increase in seafood price, (3) decrease in food availability, and (4) didn’t have enough money to buy the food they wanted. Three additional indicators were derived from questions regarding how purchasing habits changed after COVID regarding (5) type of seafood product, (6) fresh seafood purchasing location, and (7) cooked seafood purchasing location. A normalized score ranging from 0 (least impact) to 1 (most impact) was calculated for each indicator per regency. A combined COVID Impact Index was then calculated per regency by combining these seven seafood consumption impact indicators, where each indicator was weighted equally in the final score. For additional details on how each indicator was calculated, see S1 File. The data and R code for statistical analysis is available in the Appendix/online. All shapefiles for our figures with maps are taken from the UN OCHA platform for Indonesia (https://data.humdata.org/dataset/cod-ab-idn), which is available for use under a Creative Commons Attribution 4.0 International license.

All subjects in this study received a prior informed consent form at the beginning of the survey in Bahasa Indonesia, and all participants willingly agreed (with prior informed consent) to participate and to the use of data to be kept confidential and to be used for scientific purposes in the survey. All subjects provided consent to participate, by indicating that they had read the purpose and aims of the study on the first page of the survey. The research did not include any medical, sensitive or individually identifiable information for use in the analysis or publication. Participants were asked about their basic demographic information (i.e., gender, age, location, job) and seafood consumption frequency and diversity. The study did not include minors. The study design and protocol complied with and was approved by the Ethics Committee at the Leibniz Centre for Tropical Marine Research (ZMT), which standards align with the Declaration of Helsinki and the German Research Foundation.

## 3. Results

### 3.1 Survey items and descriptive statistics

Our survey was completed by 1518 respondents across 8 regencies and 2 cities in the West Nusa Tenggara province. Demographic and socio-economic survey items are shown in Table 1, and sample size and representativeness of each item are shown in S1, S2 Tables in S1 File. Responses to questions regarding seafood consumer preferences and perceived changes during COVID-19 are shown in Table 2. A majority of respondents agreed that they ate less seafood (61.24%) and that it was more expensive (66.12%) during COVID-19, compared to before. Regarding seafood availability, 37.77% agreed that the products they usually buy were less available. Nearly all respondents (94.13%) agreed that seafood is an important part of culture in the region, with 75.34% agreeing that they know the region where their seafood products come from and 89.19% preferring to buy locally produced seafood. Only 41.86% agreed that they had enough money to buy the food products they wanted during COVID-19.

**Table 1:** Demographic and socio-economic survey items and response formats.

Survey item	Response format
Age	Under 20, 20–29, 30–39, 40–49, 50–59, 60+
Gender	Male, Female
Education	Primary school, Junior high school, High / vocational high school, University +
Household size	1, 2, 3, 4, 5, 6, 7, 8, 9, 10+
Occupation	Fisherman, Fishery cultivators, Traders, Government employees, Private employees, Entrepreneurial / owning a business, Student, Housewife, Casual / daily worker, NGO workers, Teacher / lecturer, Does not work
Income per month (Before & During COVID-19)	Rp 0—Rp 1.000.000, Rp 1.000.001—Rp. 3.000.000, Rp. 3.000.001—Rp. 5.000.000, More than Rp. 5.000.000
Expenditures per month (Before & During COVID-19)	Rp 0—Rp 1.000.000, Rp 1.000.001—Rp. 3.000.000, Rp. 3.000.001—Rp. 5.000.000, More than Rp. 5.000.000
Location	Mataram Kota, Lombok Barat, Lombok Utara, Lombok Tengah, Lombok Timur, Sumbawa Barat, Sumbawa, Dompu, Kabupaten Bima, Bima Kota
Island	Lombok, Sumbawa
Development	Urban, Rural
How often do you eat fisheries products per week on average?	1–2 days, 3–4 days, 5–6 days, 7+ days

**Table 2:** Seafood consumer preferences and perceived changes during COVID-19.

Questions	Agree	Disagree	Don’t eat fish
During COVID I ate less seafood than normal.	61.24%	34.54%	4.22%
During COVID, seafood was more expensive.	66.12%	30.59%	3.29%
The seafood I usually buy or eat was not available during COVID.	37.77%	58.73%	3.49%
Seafood is an important part of food culture in Lombok.	94.13%	5.86%	NA
I know the region where my seafood comes from.	75.34%	21.03%	3.63%
I prefer to buy seafood produced locally.	89.19%	7.58%	3.23%
During COVID, I had enough money to buy the food I wanted.	41.86%	58.14%	NA

**Fig 1:**
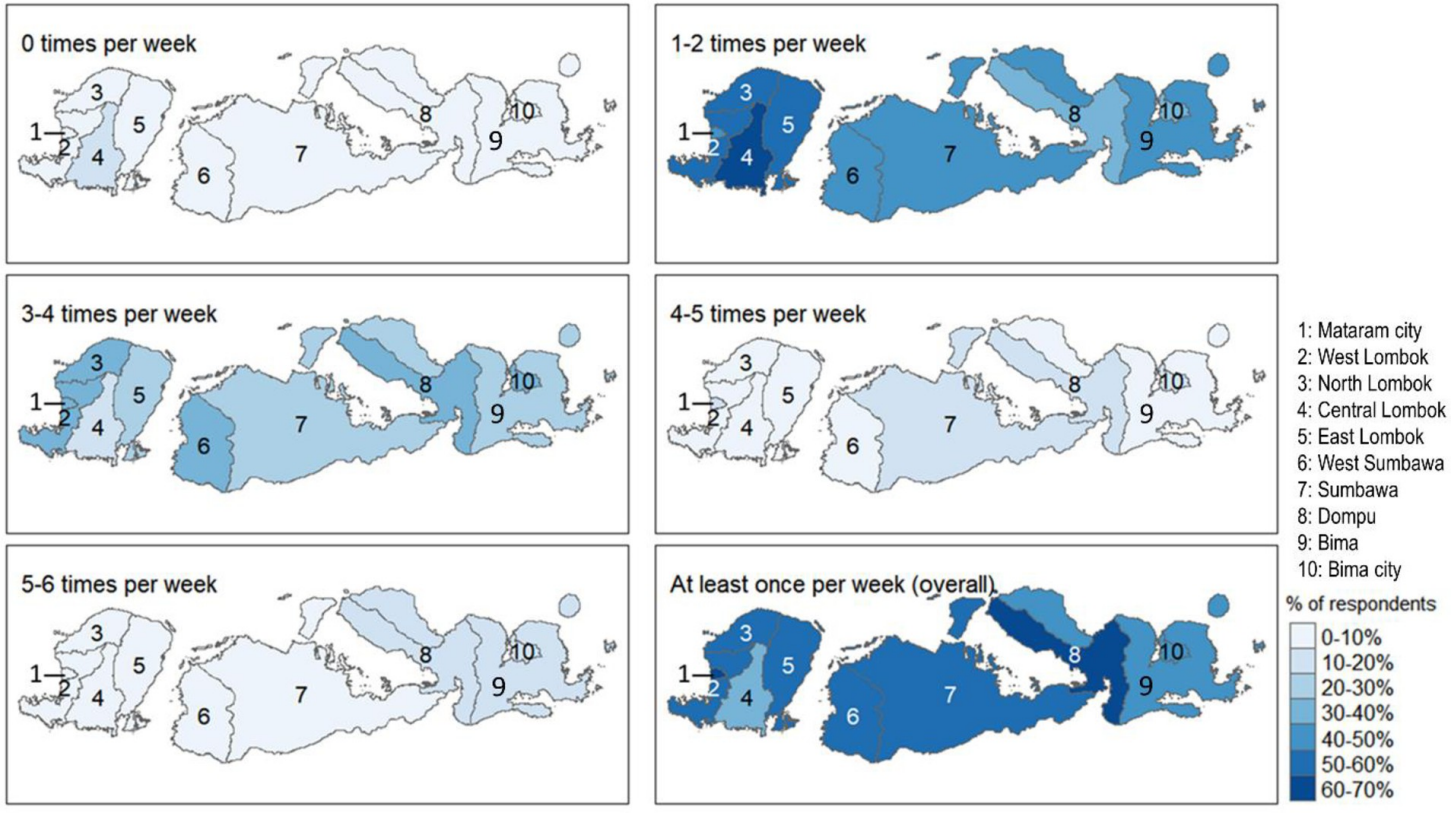
Frequency and distribution of fish consumption per week across regencies.

**Fig 2:**
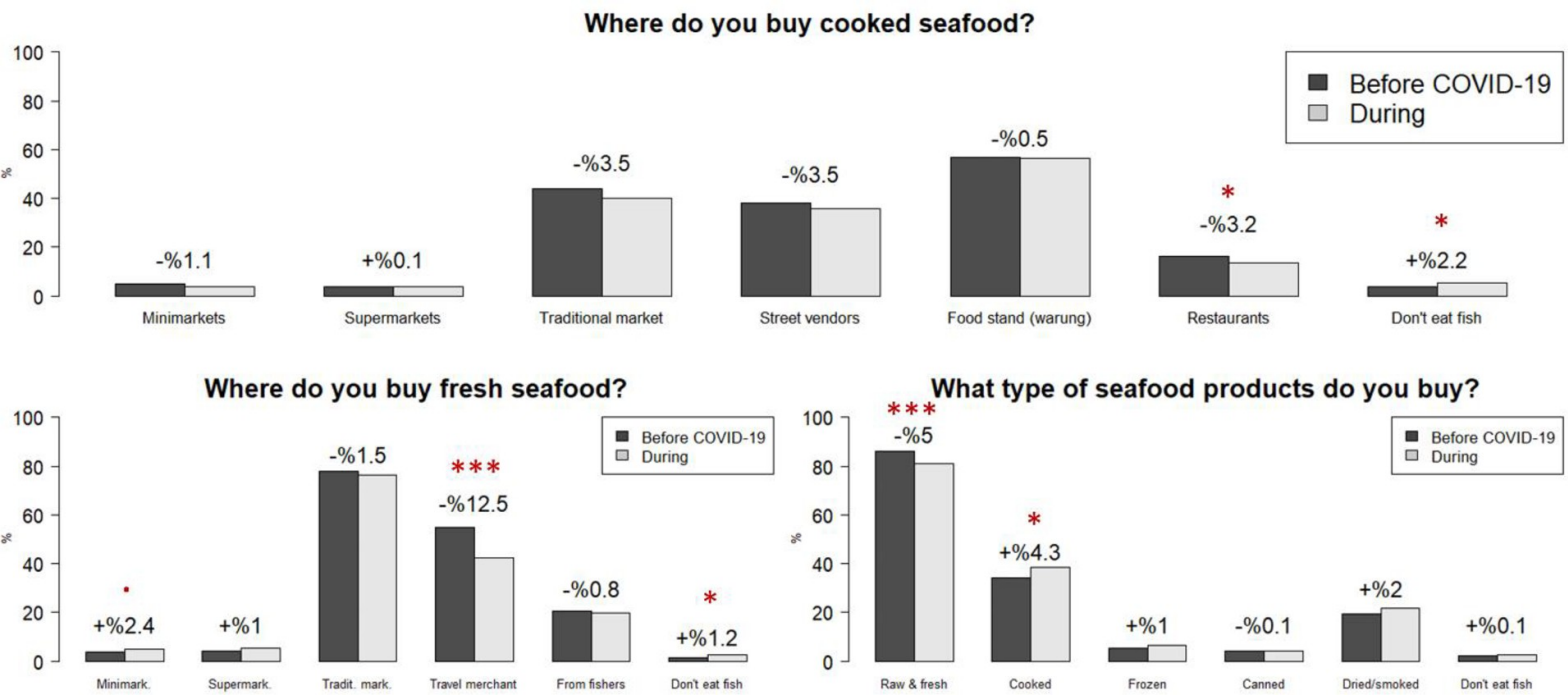
Where cooked seafood is accessed and purchased, before and during the pandemic (top). Where fresh/raw seafood is accessed and purchased (bottom left). The types of seafood products purchased (bottom right). Percentages indicate change during COVID-19. Red stars indicate the significance level of two-proportion z-tests conducted on the response changes between the two periods. Significance codes: <0.001 ‘***’ <0.01 ‘**’ <0.05 ‘*’ <0.1 ‘.’ Full response results are in the S4 Table in S1 File.

The frequency and distribution of fish consumption per week varies across the province (Fig 1), providing baseline data for the region. Dompu regency in central Sumbawa has the highest percentage of respondents (60–70%) who consume fish at least once per week. In the three east Sumbawa regencies (Dompu, Bima, Bima Kota), 20–30% of respondents eat fish 5–6 times per week, the highest in the province. At a minimum, 50–60% of respondents or higher eat fish at least once per week. The lowest rate of fish consumption is in the Central Lombok regency. Changes in where cooked and fresh seafood are purchased, as well as changes in product type during the pandemic are shown in Fig 2.

**Fig 3:**
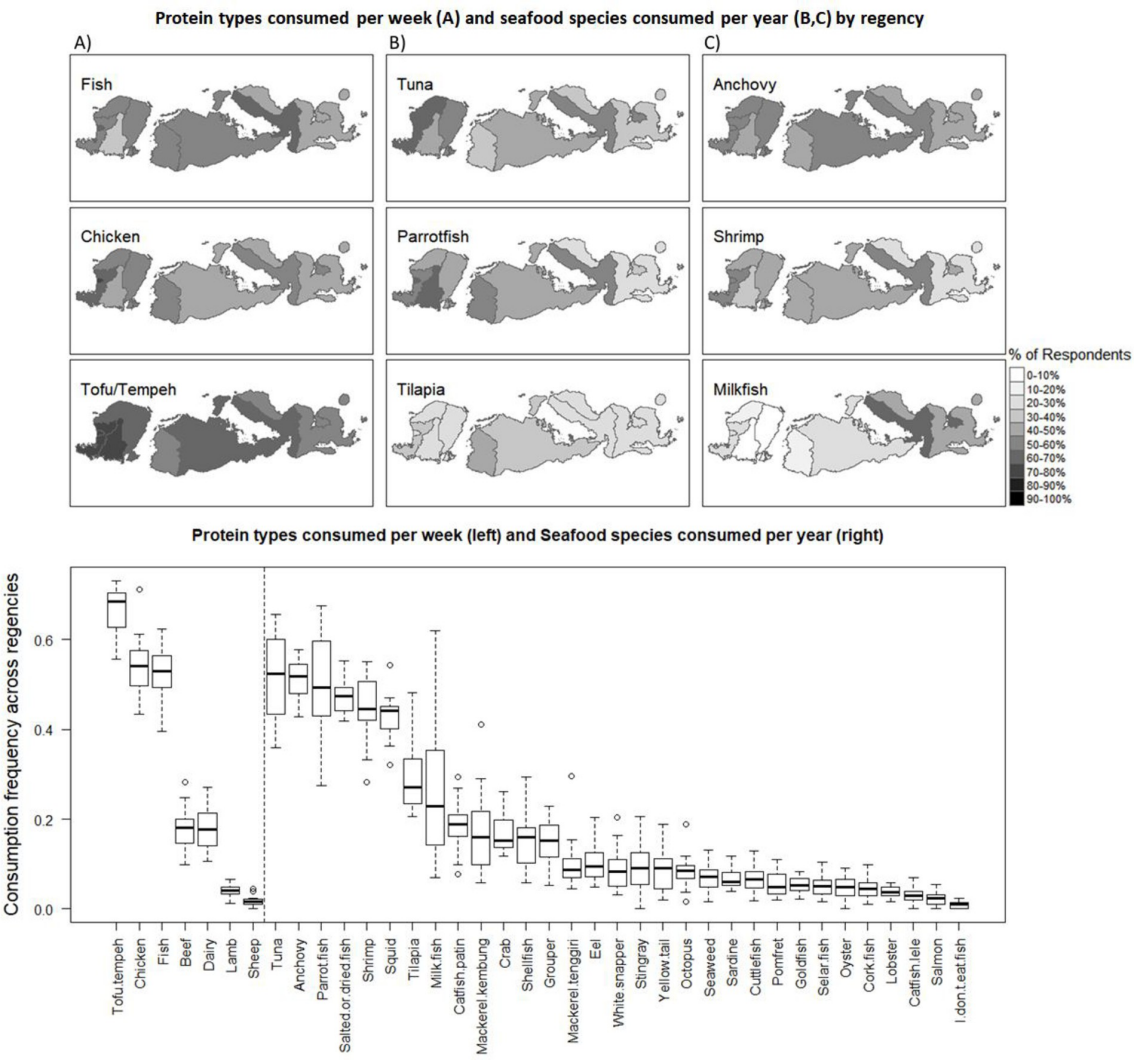
Spatial distribution of the average frequency per regency of protein types eaten per week (top A) and the average frequency per regency of 6 of the most frequently consumed seafood species per year with the highest variability across regencies (Top B,C). On the bottom, the quartile frequency distribution across regencies of each protein type per week (bottom left) and each seafood species per year (bottom right).

**Fig 4:**
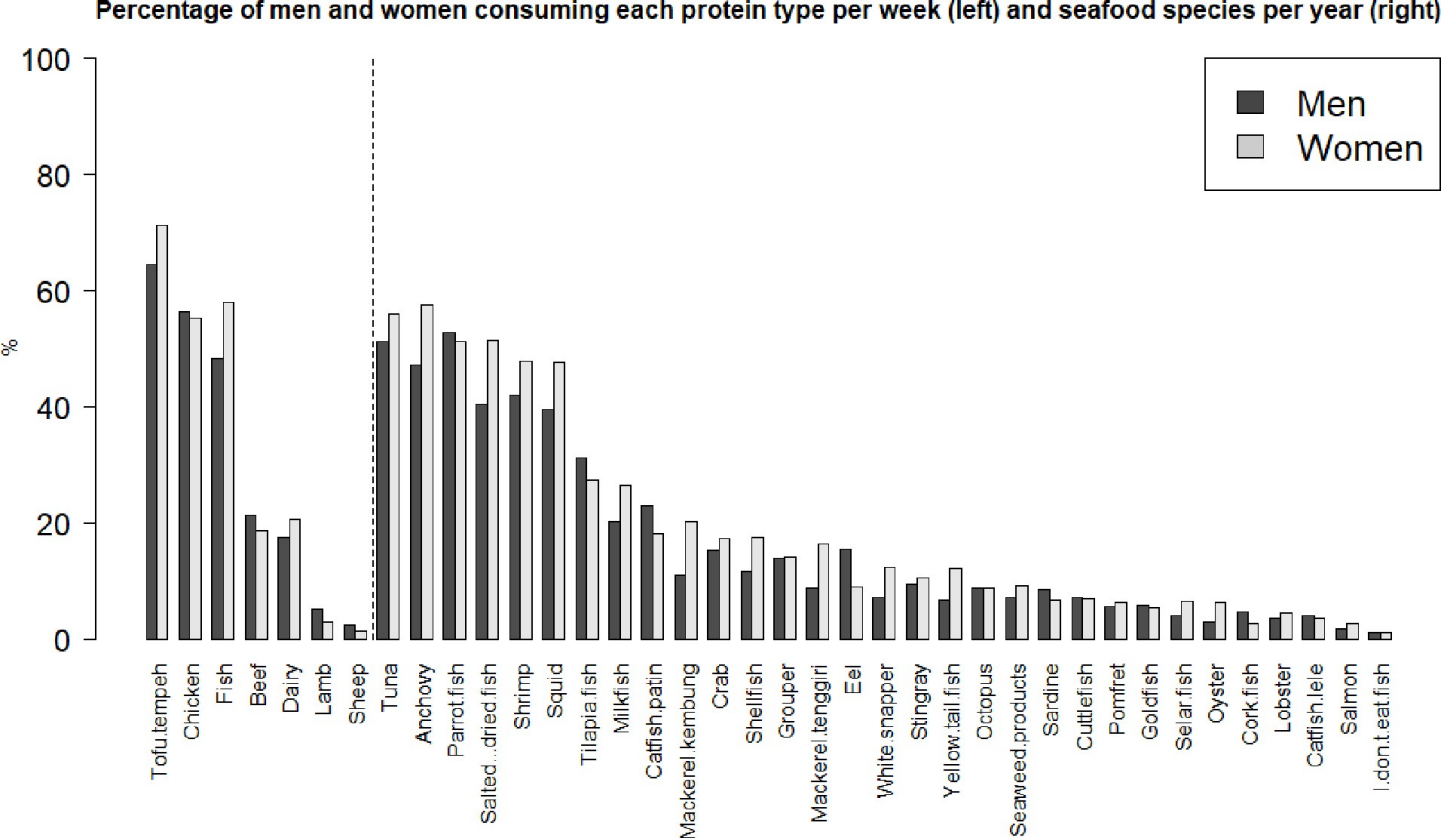
Gender ratios of protein types consumed per week (left) and seafood products consumed per year (right), sorted by the percentage of respondents in the sample population for the whole province.

The frequency and distribution of selected protein products consumed per week is shown in Fig 3. Tofu/tempeh is the most frequent in the province, with the highest frequency of per week consumption in Mataram Kota, West Lombok and Central Lombok. However, the highest diversity of seafood species consumption per year is in Mataram Kota (6.74), Dompu (6.33) and West Lombok (6.28), with the lowest in Bima (4.14) (S9 Table in S1 File). Chicken and fish have similar consumption frequencies in the province, the second and third highest after tofu/tempeh. The highest percentage of respondents consuming fish are in Dompu and Mataram Kota, the lowest in Central Lombok. Tuna, anchovy and parrotfish and the most frequently consumed seafood species, followed by shrimp, tilapia and milkfish. The frequency of respondents who consume each species differs for each species by regency. For example, tuna is more frequently consumed by respondents in North Lombok, parrotfish in Central Lombok, tilapia in West Sumbawa and milkfish in Dompu. The largest difference in protein type consumption per week between men and women is fish, as 10% more of the female population eats fish at least once per week, and ~7% more tofu/tempeh (Fig 4). Men eat slightly more beef, lamb, chicken and sheep. Women consume a median of 5 and mean of 5.82 different seafood species per year (Fig 4). Men consume a median of 4 and mean 5.10 different seafood species per year. Respondents in the highest education classification (University or higher) eat on average more seafood species diversity per year at 6.43, compared to the lowest income level at 2.12 (Primary school), 3.04 (Junior high) and 4.62 (High school) (S8 Table in S1 File).

### 3.2 Statistical analysis

ANOVA tests were run to test for differences in mean number of seafood species consumed per year (see Figs 3 and 4 for list of species) with spatial and socio-economic factors from the descriptive data. Results indicate that the location of respondents was significantly related to the number of seafood species consumed (F(9,1507) = 6.252, p = 1.04e-08). Tukey Honest Significant Differences (HSD) tests showed that number of species consumed was significantly different particularly between Kota Mataram, the region with the highest mean number of species consumed (6.74), and the regencies of Central Lombok (4.35), East Lombok (4.70), and Bima (4.15). Analysis also indicated that respondents with higher income consumed a higher mean number of species than lower income groups (F(3,1513) = 61.46, p = <2e-16), with Tukey HSD tests showing significant pairwise differences between all but the two highest income groups. A similar pattern was found regarding education level and species consumed (F(3,1513) = 28.06, p = <2e-16), with higher education level corresponding to a higher mean number of species consumed. Full results of pairwise Tukey HSD tests can be found in the Section 1 in S1 File.

This survey provides a baseline study for where seafood is accessed/purchased and the types of seafood products that are consumed in the province, as well as findings on changes during COVID-19. Over 85% of respondents consume raw or fresh seafood products, with a -5% decline during the pandemic (Fig 2). Two-proportions z-tests were calculated to test for significant differences in seafood buying preferences before and during COVID-19 (Fig 2). Descriptive data is shown in Fig 2, comparing response frequencies of where they purchased cooked seafood, fresh seafood, and what types of seafood products they buy. The percentage of respondents purchasing cooked seafood from traditional markets decreased during COVID-19 with a p-value slightly over 0.05 (43.8% to 40.3%, p = 0.051), while purchases of cooked seafood from restaurants also decreased significantly from 16.2% to 13.4% (p = 0.036). Regarding fresh seafood, purchases from traveling merchants were most significantly impacted, decreasing from 55% before COVID-19 to 42.4% during (p<0.001). Purchasing preferences for seafood product type showed a clear pattern. Raw/fresh seafood purchases decreased from 86% to 81% (p<0.001), while cooked seafood purchases increased from 34.2% to 38.5% (p = 0.016).

Logistic regression models were fit to the COVID-19 change survey questions as dependent binary variables (i.e., agree or disagree), to test model fit for socio-economic and demographic factor significance (Table 3). During COVID-19, the amount of fish an individual eats per week in general is a significant factor in explaining whether they ate less fish during the pandemic, with those eating fish 3–4 times per week (0.009) more likely to agree that they ate less during the pandemic. On Sumbawa island, respondents were significantly more likely to agree (0.03) that the fisheries products they usually buy were not available during the pandemic, as well as with those respondents whose income went up (0.04). The oldest segment of the population (i.e., 60+) were significantly more likely to agree (0.005) that fisheries products are an important part of the culture in the region, and also agree (0.047) that they know where their fisheries products come from. Respondents who eat more fish per week (i.e., 3–4 times per week or more) are significantly more likely to agree (>0.000) that they buy local seafood produced in the region, especially if they are in the 30–39 or 60+ age group (>0.000). Lastly, males were significantly more likely to agree (0.017) that they had enough money to buy the food that they wanted during the pandemic.

**Table 3:** Coefficients of the logistic regression models of the demographic and socioeconomic factors associated with seafood consumption change questions during COVID-19.

Coefficients	Estimate	Stand. error	t-value	Pr(>|t|)	Signif.
**During COVID I ate less fisheries products than normal.**					
(Intercept)	1.5072	0.4096	3.680	0.01430	*
Eat fish 1–2 times per week	0.7721	0.5478	1.410	0.21771	
Eat fish 3–4 times per week	-2.0586	0.4989	-4.126	0.00912	**
Eat fish 5–6 times per week	-1.5773	0.5050	-3.123	0.02615	*
Eat fish 7+ times per week	-0.9689	0.4250	-2.280	0.07158	.
**The fisheries products I usually buy or eat were not available during COVID.**					
(Intercept)	-1.7987	0.3143	-5.723	0.00228	**
Gender: Male	1.3197	0.5655	2.334	0.06689	.
Island: Sumbawa	1.7889	0.5935	3.014	0.02961	*
Income: went down	0.7368	0.5095	1.446	0.20778	
Income: went up	2.9452	1.0911	2.699	0.04282	*
**I prefer to buy fisheries products produced from West Nusa Tenggara.**					
(Intercept)	-0.28084	0.60594	-0.463	0.654021	
Eat fish 1–2 times per week	1.18149	0.62206	1.899	0.089986	.
Eat fish 3–4 times per week	2.97580	0.57288	5.194	0.000568	***
Eat fish 5–6 times per week	2.16807	0.43799	4.950	0.000791	***
Eat fish 7+ times per week	18.77537	0.83206	22.565	3.12e-09	***
Age: 20–29	-0.05361	0.96442	-0.056	0.956885	
Age: 30–39	3.28369	1.27954	2.566	0.030371	*
Age: 40–49	1.47878	0.84130	1.758	0.112664	
Age: 50–59	2.47010	1.14848	2.151	0.059962	.
Age: 60+	20.23082	1.44534	13.997	2.05e-07	***

Significance codes: 0

‘***’ 0.001

‘**’ 0.01

‘*’ 0.05 ‘.’

### 3.3 Covid-19 impact index on seafood consumption at regency level

**Fig 5:**
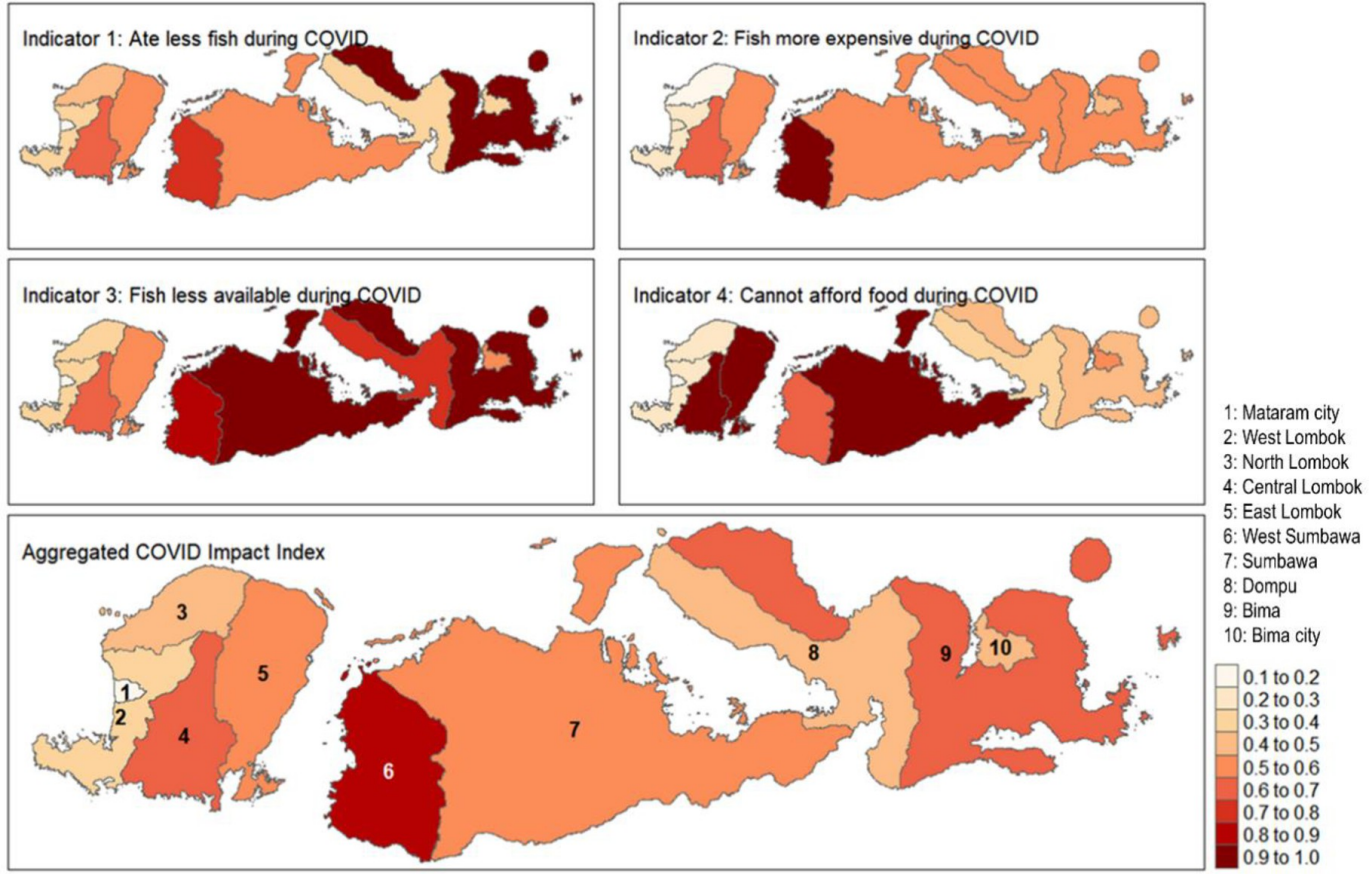
COVID seafood consumption impact index scores by regency/*kota*. Four (of seven total) indicators along with aggregated index score.

The COVID seafood consumption impact index scores are shown by regency (*kabupaten*) and city (*kota*), representing relative differences between regencies in terms of reported seafood consumption impacts due to COVID-19 (Fig 5, scores in S6 Table in S1 File). West Sumbawa had the highest aggregated impact index score (0.806), and Kota Mataram the lowest (0.169). There was observable heterogeneity in terms of specific impacts across districts between the seven indicators used to calculate the aggregated index, but clear patterns can be observed. Kota Mataram had the lowest score across 3 indicators and scored relatively low in others, while Bima and West Sumbawa regencies each had the highest impact score in two indicators (S6 Table in S1 File). Full documentation on how the index was calculated can be found in the Section 2 in S1 File.

## 4. Discussion

Our findings provide a regency level baseline for seafood consumption patterns in the province of West Nusa Tenggara, and suggest that COVID-19 has inflicted heterogeneous changes in the access and consumption of seafood across regencies, which are substantial in some regencies including West Sumbawa and Central Lombok. As a baseline, our findings show that the composition of protein types and seafood species differs across West Nusa Tenggara, especially between urban and rural regencies. Rural regencies incurred more changes in consumption patterns due to the pandemic, which are likely related to access and income shown below. This result differs from a study examining food consumption changes between urban and rural areas across Indonesia from 1976 until 2013, which used time series survey data on 300,000 households collected by the Indonesian National Statistics Office. The national time series analysis, conducted by Putra et al., [45], indicates a declining share of food expenditures in urban areas compared to rural areas, which is common as income increases. However, they found that food composition in urban areas remained stable, considering all food types, likely due to lower-income allocated to food and higher non-food expenditures of urban inhabitants. Observed differences in seafood consumption composition in our study are likely due to region specific differences, or perhaps a trend that is only apparent when analyzing protein sources or seafood in isolation.

Results from the COVID seafood consumption impact index identified Kota Mataram, the largest municipal region in the province, as scoring consistently low (i.e., less impacted) across indicators, while more sparsely populated rural regencies such as West Sumbawa and Bima had the highest impact scores (S6 Table in S1 File). Urban-rural spatial differences may be related to market and access diversity being lower in rural areas (e.g., further away or fewer options). Access to seafood at traditional fresh fish markets was likely restricted during COVID-19 lockdowns. For example, our author group observed closures of fresh seafood markets in the region’s largest city Mataram during this time. More broadly, our findings suggest a significant decrease in fresh seafood consumption during the pandemic, but urban areas are likely to have access to other market types for seafood and more diverse products, whereas rural areas may not. Our findings suggest this trend with significant declines in seafood access at traditional markets and through traveling merchants, and increasing access in modern mini- and supermarkets. Similar findings were shown by Paganini et al., [46] who showed that Javanese farmers reported using traditional food markets such as local vendors and farmer’s markets 5 percent less frequently during the first three months of the COVID-19 lockdown. The authors further found price increases of 50% or more for all foods, and that fruit consumption, along with fish and meat, decreased. Another study by Gibson and Olivia [47], conducted using the Indonesian Family Life Survey data on 3,951 households, indicated that the distance from the provincial capital and the quality of the road infrastructure and electricity was significantly related to rural household income and the number of small businesses. In relation to seafood, this means more potential for food markets to have regular easy access to fresh fish and a higher potential for refrigeration.

Our baseline findings, which indicate differing seafood composition in rural areas, are likely due to a variety of reasons such as income, access, culture, local production (i.e., aquaculture) and storage potential. If supply and value chains for seafood stagnate, particularly for traveling merchants as shown in our analysis, and refrigeration of local fish is not possible, then maintaining regular seafood consumption in rural areas during the pandemic mobility restrictions would be difficult unless it is produced regularly on-site. Restricted access likely raised prices, even if seafood products were regularly available. Another reason may be that rural inhabitants are more likely to have personal food gardens for self-sufficiency, which can replace purchased food items if they become scarce or more expensive [48]. In contrast, urban residents may have exhibited more consistent composition of seafood consumption, but their adaptive mechanism is different, rather switching the type of market to search for lower prices. In an additional study utilizing the Indonesian Family Life survey data, Colozza and Avendano [49] show that the acquisition of fish is consistently higher for rural residents (non-Jakarta), and that urban residence is significantly associated with lower household expenditure shares for fish.

Looking forward in regards to the longer lasting impacts of COVID-19, findings by Belton et al., [24] in a longitudinal study throughout 2020 of 768 respondents in Bangladesh, Egypt, India, Myanmar and Nigeria, showed that impacts on demand for aquatic foods have been longer lasting than impacts on their supply. This may suggest that once consumer behavioral patterns in relation to seafood changed due to the pandemic, they may remain so for longer periods or not return to pre-pandemic patterns. While many studies have been recently published on the impacts of COVID-19 on seafood supply chains and distribution networks [23, 33, 36], few studies have examined consumer level consumption patterns at larger scales. A study in the United States by White et al., [26], using online search string data and published non-scientific media content at the national level, indicated that the mean number of people visiting fish and seafood markets decreased by 30% in 2020 and searches for “seafood restaurant” declined by approximately 70%. In contrast, a study by Costa and colleagues [50] reviewed the rise of online platforms–WhatsApp, Facebook, new apps and websites—for direct-to-consumer sales of fisheries products during the pandemic, which aimed to shorten supply chains and increase access. They found 122 initiatives in 48 countries including Indonesia, but few details are known about the success or degree of popularity of e-commerce initiatives for fisheries products in the country other than they are not yet a widespread practice because many fisheries products are considered traditional foods that are sold in traditional markets [51]. Indonesian seafood consumption patterns have unique histories and cultural origins. Learning across contexts to examine changes in seafood consumption due to COVID is generally useful for hypothesizing relevant drivers and pathways of change, examined further below. However, further context specific data and analyses are needed in Indonesia to draw conclusions that may be relevant to local policy recommendations across the urban-rural divide.

We observed gender differences in baseline seafood consumption patterns. Fish products, particularly raw and freshly cooked, are the highest source of animal protein in the province (nearly equal with chicken), especially for women. More women (+10%) consume seafood than men, and women have a more diverse seafood diet on average. One explanation may be that women prefer more seafood due to awareness of dietary health benefits, or that seafood products tend to be cheaper sources of animal protein than red meat products. A recent study by Gibson et al., [52] based on a survey of 66 households with mothers and children in small-scale fisheries in Komodo, Indonesia, found that fish are a primary source of animal-protein with essential nutrients for both mothers and children, but that both groups fell below the minimum recommendation for a diet with micronutrient adequacy, with delays in fish consumption for young children. The study further discusses the findings in relation to the challenges for increasingly nutritious seafood consumption for women and children to meet nutrition goals by increasing awareness of the benefits, improving access fish and other food diversity and reducing misinformation and taboos about seafood consumption. The role of reducing myths and/or misinformation about fish that deters consumption was also a top driver of the amount of fish Indonesians choose to eat in a study by Wijaya et al., [53] with 427 respondents in Yogyakarta and Central Java. Overall, pandemic related impacts on seafood access may further increase challenges for sufficient access to nutritious seafood access, especially in rural and low income groups. However, the challenges may be embedded in cultural norms, where there are many potential reasons why people make certain food choices.

Our findings also indicate that men eat more red meat products (i.e., beef, lamb, sheep) than women, and were more likely to agree that they had enough money to buy the food products they prefer during COVID-19. Women earn less income than men, on average in our study, although this gap became smaller during the pandemic. Men may eat food outside of the home more often, whereas women eat more home cooked food. Men were more likely to agree they had enough money to buy the food they wanted. An explanation may be that the seafood products men buy may have different prices than the ones that women prefer or men may have more flexible preferences. Men were slightly more likely, although not significant, to agree that the seafood products they prefer were not available during COVID-19. Further studies are needed to explore these speculative hypotheses in the context of Indonesian gender and household purchasing and consumption dynamics, which we encourage.

Seafood options (e.g., the number of species regularly consumed per year), may be associated with more local or traditional food recipes than other animal protein products. Arsil et al., [54] note that Indonesian consumers believe that local foods have higher quality and cheaper prices than national and imported foods, and that buying local foods is associated with a positive narrative around villages, local production and small-scale. Consumers buying local seafood may not have been as impacted as consumers who prefer to buy seafood in larger supermarkets sourced beyond local areas due to stronger reliance on supply chains. Our study shows a strong preference for traditional and locally oriented locations for purchasing seafood which may be a reason why minimal changes in overall seafood consumption were observed. Seafood is, especially through aquaculture of milkfish and shrimp [55, 56], a large part of the culture dietary composition in West Nusa Tenggara, and when sourced locally, may be more resilient to supply chain closures and production interruptions faced elsewhere during the pandemic. However, substantial amounts of seafood produced such as tuna, lobster, snapper and shrimp is also exported outside the province, which can lead to adverse supply chain effects. A study by Campbell and colleagues suggests that the active number of small-scale fishers and traders dropped by over 90% in the first year of the pandemic in south Sulawesi, Indonesia. An additional study by Bassett et al., [36] indicated that halted exports of fish to China from North Sumatra in the early pandemic flooded local markets with fish, increasing community access, but lowered prices below sustainable livelihood levels for local fishers. No studies, to our knowledge, have examined such trends in West Nusa Tenggara.

Those who eat more fish per week were more likely to agree that they ate less fish during the pandemic, with the ‘3–4 times per week’ group having the highest significance. Those eating more fish per week (7+ times) perhaps changed less due to the pandemic because it is a regular part of the diet, whereas occasional eaters (3–4 times per week) may be willing to be more flexible if availability decreases. Similarly, those eating fish more often, were also more likely to agree that they buy fish products from the region, along with the oldest respondent group (age 60+). More frequent eaters may be more aware of where their food is sourced, which may be linked to quality preferences or behavioral patterns for purchasing linked to local markets serving locally produced seafood established when they were younger (i.e., for older groups), when fewer market options were available. Local seafood may be fresher and higher quality than imported products, or high frequency consumers may be more closely connected to local fishing or aquaculture producing communities or their traders, thus they consume more and prefer to support local communities and businesses. A study by Widayat and Arifin [57], surveying 147 Indonesian adolescents and their drivers of food choices during COVID-19, indicated that attitudes related to the pandemic are a good predicator of food purchasing behavior. Younger individuals were less likely to purchase imported food, more likely to purchase food online and avoid the use of cash if they believed strongly in the negative health impacts of virus infection. A possible explanation of our consumption frequency findings and the observed relationship to reduced fish consumption during the pandemic may be that younger individuals consume less fish overall and may be more educated about the negative impacts of COVID-19 infection, and therefore more likely to avoid fish products (especially at local in-person markets) compared to older individuals who may be less informed and/or source fish from locally with difficult to change behavioral routines for shopping and eating. Our findings suggest that older respondents (60+) are more likely to agree the seafood is an important part of the local culture and that they know where the seafood they buy comes from. This may suggest a strong link from older respondents to seafood producing communities, particularly in rural areas.

**Fig 6:**
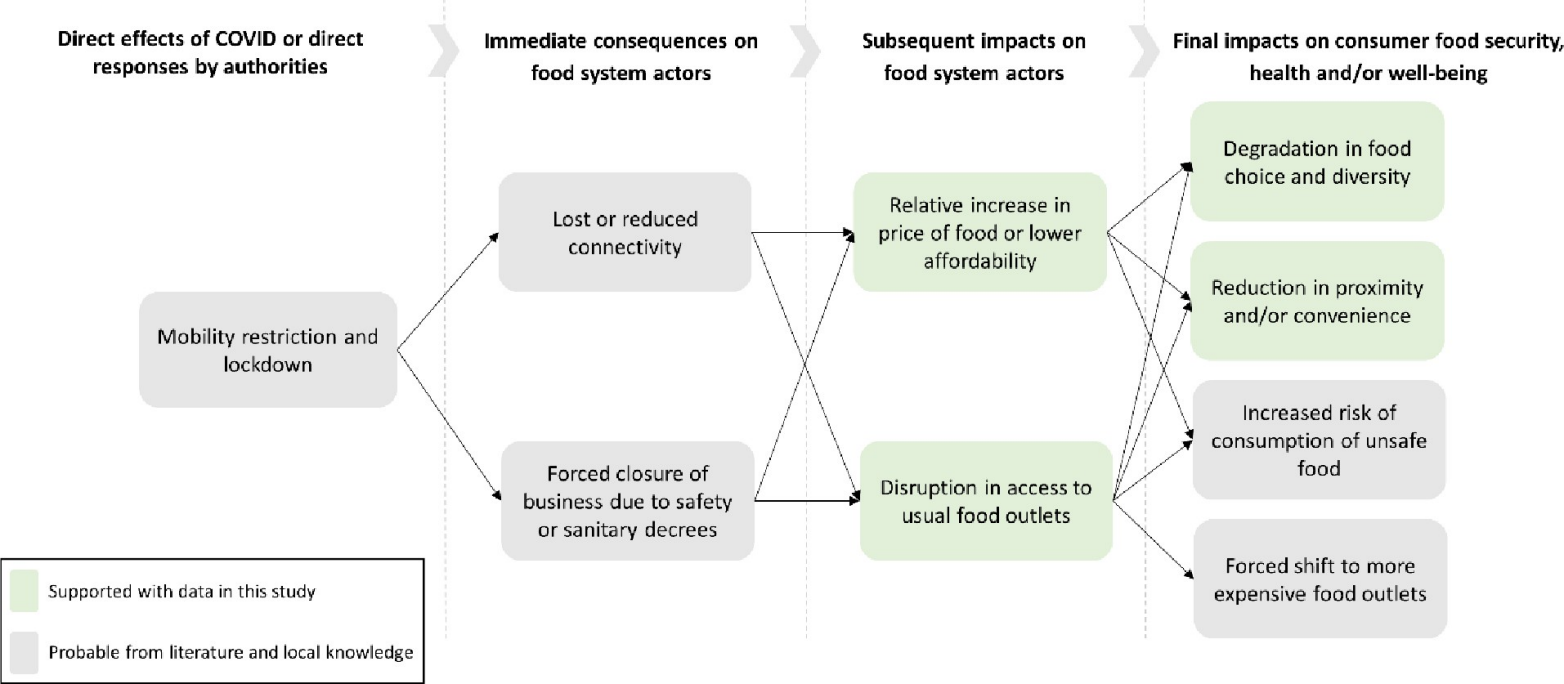
Likely impact pathways for seafood consumers during COVID-19 in West Nusa Tenggara, Indonesia. Pathway categories and flow based on the global literature of empirically derived impact pathways on food systems during COVID-19 by Béné et al., [20]. Displayed categories are only those relevant for this study context. Boxes colored green reflect impacts supported by the analysis in this study. grey boxes are speculated based on the local knowledge of the authors and literature.

The specific findings of our study in isolation do not provide direct evidence of the potential impact pathways by which the pandemic effected consumers through a series of likely causal events. To examine this potential impact pathway with support from our findings, we draw on the empirically based conceptual framework developed by Béné et al., [20]. Their framework is based on a global review of 337 documents covering 62 countries on the potential impact pathways on food systems from COVID-19 during 2020. We assessed the relevant framework components for our case, and created a context relevant impact pathway diagram (Fig 6). The impact pathways include where our data contribute directly and what is more speculative based on our local knowledge and the literature (Fig 6). The primary direct effect of the pandemic assessed in this study is mobility restrictions due to both government policy and fear of infection. This likely led to either closure of seafood access points or at a minimum reduced connectivity in seafood value chains including distribution beyond local production areas to final consumer markets. These disturbances can lead to disruptions in access, increases in price or decreases in product quality. Consumers face the final impact of choice reduction (in our case, eating less seafood or none at all), loss of convenience, increased risk of unsafe food and/or are forced to shift to more expensive outlets. These impact pathways attempt to assess the logical series of events back to direct driver, in this case mobility and economic restriction policies. Importantly, these impact pathways are almost certainly not fully comprehensive. There are likely other potential reasons why consumer seafood choices differ that are yet to be explored, including the interaction of food systems with our critical aspects of society such as the role of social media, economic fluctuations or demographic changes which this study has not explored. Nonetheless, impact pathway analysis is a useful step towards unpacking causal mechanisms in food systems, particularly their value chains, and their transformation due to disturbances and societal change.

More broadly, understanding changes and transformations in food systems is a critical step towards understanding how and why human nutrition and wellbeing can be achieved more sustainably [58, 59]. Aquatic food systems have substantial potential to continue contributing to sustainable healthy diets in many world regions [60–62], particularly through the rapid growth of aquaculture in countries such as Indonesia [63, 64]. Substantial effort has been put into understanding the production of seafood [65, 66], their corresponding value chains [67] and governance [68, 69]. However, less is known about changing consumer preferences in top seafood producing countries such as Indonesia during the pandemic and now after, and how those changes may be reciprocally effecting value chains, production and human health. Such issues will need continued research and governance engagement to meet local needs and sustainability aspirations as envisioned in the Indonesian national strategy for transforming the blue economy [70].

### 4.1 Methodological reflections

Our sample population is skewed towards younger and more educated respondents, however, our statistical analyses were weighted to account for this. Although our sampling method was rapid and safe during COVID-19, due to being entirely digital which was the only ethically responsible way to collect new data during this time, segments of the population with minimal or no smart phone access may have been excluded. This is more likely to be a rural low income segment of the population. Our findings suggest that rural areas incurred more changes, and this may even be underestimated given our sampling procedure. We utilized a IP address restriction software, available in Limesurvey, that only allows survey submissions from one IP address. We did this to mitigate multiple submission from the same device, because we offered a small financial incentive (~0.67 Euro phone credit) to all individuals completing surveys. Without IP restrictions, individuals could submit multiple responses with false data to receive the phone credit. However, limiting each device to one submission may have not enabled groups who share a device (e.g., a family or couple with one phone or computer) to submit multiple real entries. This is a methodological trade-off in our view. Restricting IP address reduces the false data entry potential but could limit real entries among groups with shared devices, while without IP restriction would be more inclusive to groups with shared devices but risk multiple false data entries. We decided to restrict IP address under the decision that reducing false entries (i.e., knowing that your data is real) was more important than not restricting for group inclusion. Mobile device penetration in Indonesia is very high, and our sampling strategy produced a data set that enabled a weighted analysis across stratified demographic groups.

In regards to our platform usage sampling strategy through WhatsApp and Facebook, also used by Campbell and colleagues [38], they have an estimated 87% and 85% penetration rate in the country respectively as of 2020 (https://www.statista.com/statistics/284437/indonesia-social-network-penetration/). This study has shown online surveys distributed on WhatsApp can rapidly obtain large sample sizes to examine disruptions during COVID-19 or other disaster events in a safe way in less developed regions. There is an opportunity to apply the same methodology on other COVID-19 impact topics such as on seafood production as well as in other provinces of Indonesia and throughout the global tropics where high online and mobile device penetration can provide reasonably representative reasonably sampling opportunities.

We also acknowledge that the framing of our questions and response format in the survey have potential for bias. A core goal of this project was to implement a rapid assessment survey instrument in the context of the rapidly changing social, economic and political context of COVID-19 infection rates and policy strategies at the time. In doing so, we opted for quick response formats (i.e., agree or disagree) for question numbers 10–15 in our survey (see S1 File), to optimize response rates in a short period. Even with a locally substantial financial incentive for completing the survey, which took an average of less than 5 minutes to complete, our complete response rate was 63.7%, with 1517 complete response and 862 incomplete responses out of 2379 people who opened the survey link. This was during a period of five weeks, where we believe without a brief survey that reduces cognitive burden (and this incentivizes completion), and an ads campaign sampling strategy, we would have been sampling across a much longer time frame that would then risk changing social, economic and political circumstances across early and late response entries in the population. There are additional potential biases in the framing the questions. We adopted a response format on survey questions 10–12 where the ‘agree’ answer would provide data supporting our hypothesis, rather than a ‘disagree’ framing. This could be critiqued as leading the respondents towards confirmation of our hypothesis, where a more nuanced response format (e.g., strongly agree, agree, neutral, disagree, strongly disagree) may have provide more variation or insight into the trends.

## 5. Conclusion

Seafood consumption patterns in West Nusa Tenggara, Indonesia vary by regency, with observable differences between gender, education and age, as well as in product types, market types and species diversity. COVID-19 has significantly changed some of these patterns, including less seafood consumption in restaurants, more people who don’t eat fish, less seafood purchases from traveling merchants, less consumption of raw or fresh seafood, increases in cooked seafood consumption, overall less seafood consumption and overall increased prices of seafood during the survey period. Our findings provide a baseline for understanding the impacts of such food security related disasters. Fisheries products in Indonesia are essential for food security, and without baseline data on consumption patterns and changes incurred due to large scale disruptions or increased growth rates in production locally, our ability to understand the role that aquatic or blue food can play in transitions toward sustainable food systems will be impaired. In particular, the rise of aquaculture and how it might be changing seafood consumption patterns and access to food requires further studies, especially in rural and low income areas where access to safe and reliable food is critical for sustainable development.

## Supporting information

S1 File (DOCX)

S2 File (R)

S3 File (DOCX)

S1 Data (XLSX)

S2 Data (CSV)
