# Supplementary material for: Seafood consumption changes and COVID-19 impact index in West Nusa Tenggara, Indonesia
Source: PLoS One. 2023 Jan 18;18(1):e0280134. doi: 10.1371/journal.pone.0280134 (PMC9847987; doi:10.1371/journal.pone.0280134)
Supplement: S1 File — (DOCX) [file pone.0280134.s001.docx]

**SUPPLEMENTARY MATERIAL**

**Seafood consumption before and during COVID-19 in West Nusa Tenggara, Indonesia**

Stefan Partelow, Ben Nagel, Adiska Octa Paramita, Nurliah Buhari

1. Leibniz Centre for Tropical Marine Research (ZMT), Bremen, Germany
2. Jacobs University, Bremen, Germany
3. University of Mataram, Mataram, Indonesia

**Section 1: Methods and results extension**


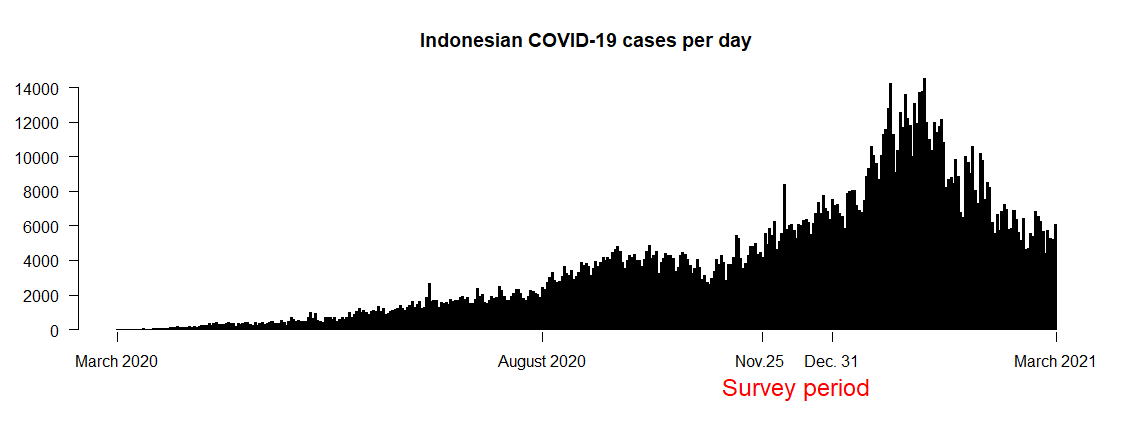


**Figure S1.** Number of new COVID-19 cases per day and the survey period for this study.

**Table S1.** Sample sizes and skews of sample-to-population in all attribute categories.

| **Data** | **Population** | **Sample** | **Sample skew** |
| --- | --- | --- | --- |
| Male | 2,461,652 | 696 | -2.67% |
| Female | 2,608,733 | 821 | +2.67% |
|  | | | |
| Under 20 | 448,927 | 240 | +3.32% |
| 20-29 | 834,811 | 668 | +20.79% |
| 30-39 | 755,283 | 343 | +1.58% |
| 40-49 | 655,459 | 172 | -6.91% |
| 50-59 | 467,565 | 78 | -7.88% |
| 60+ | 429,738 | 16 | -10.91% |
|  | | | |
| Primary school | 1,109,676 | 25 | -44.84% |
| Junior high | 365,208 | 83 | -9.83% |
| High school | 613,728 | 576 | +12.26% |
| University or higher | 298,424 | 833 | +42.41% |

**Table S2**. Sample population sizes and skews by regency.

| **Regency** | **Island** | **Urban** | **Population** | **Sample** | **Sample skew** |
| --- | --- | --- | --- | --- | --- |
| (1) Mataram Kota | Lombok | Yes | 486715 | 298 | +10.04% |
| (2) Lombok Barat | Lombok | No | 694985 | 201 | -0.46% |
| (3) Lombok Utara | Lombok | No | 220412 | 85 | +1.26% |
| (4) Lombok Tengah | Lombok | No | 947488 | 187 | -6.36% |
| (5) Lombok Timur | Lombok | No | 1200612 | 244 | -7.59% |
| (6) Sumbawa Barat | Sumbawa | No | 148606 | 52 | +0.50% |
| (7) Sumbawa | Sumbawa | No | 457671 | 166 | +1.92% |
| (8) Dompu | Sumbawa | No | 252288 | 92 | +1.09% |
| (9) Kabupatan Bima | Sumbawa | No | 488577 | 131 | -1.00% |
| (10) Bima Kota | Sumbawa | Yes | 173031 | 61 | +0.61% |

**Table S3.** Seafood consumer preferences and perceived changes during COVID-19, raw totals and percentages.

| **Questions** | **Agree** | **Disagree** | **Don't eat fish** |
| --- | --- | --- | --- |
| During COVID I ate less seafood than normal. | 929 | 524 | 64 |
|  | 61.24% | 34.54% | 4.22% |
| During COVID, seafood was more expensive. | 1003 | 464 | 50 |
|  | 66.12% | 30.59% | 3.29% |
| The seafood I usually buy or eat was not available during COVID. | 573 | 891 | 53 |
|  | 37.77% | 58.73% | 3.49% |
| Seafood is an important part of food culture in Lombok. | 1428 | 89 | NA |
|  | 94.13% | 5.86% |  |
| I know the region where my seafood comes from. | 1143 | 319 | 55 |
|  | 75.34% | 21.03% | 3.63% |
| I prefer to buy seafood produced in Lombok. | 1353 | 115 | 49 |
|  | 89.19% | 7.58% | 3.23% |
| During COVID, I had enough money to buy the food I wanted. | 635 | 882 | NA |
|  | 41.86% | 58.14% |  |

**Table S4.** Full results table for the two-proportion z-test.

| Seafood purchasing preference | Proportion of respondents before and during COVID-19 | | | Two-proportion z-test (two-tailed) | |
| --- | --- | --- | --- | --- | --- |
| **Cooked Seafood** | **Before** | **During** | **% change** | **P-value** | **Signif.** |
| Minimarket | 4.88% | 3.76% | -1.12% | 0.153 |  |
| Supermarket | 3.76% | 3.82% | 0.07% | 1.000 |  |
| Traditional market | 43.84% | 40.28% | -3.56% | 0.051 | . |
| Street vendors | 38.23% | 35.93% | -2.31% | 0.201 |  |
| Food stand (warung) | 56.95% | 56.49% | -0.46% | 0.826 |  |
| Restaurant | 16.15% | 13.38% | -2.77% | 0.036 | * |
| Don’t consume/purchase | 3.63% | 5.47% | 1.85% | 0.019 | * |
| **Fresh Seafood** | **Before** | **During** | **% change** | **P-value** | **Signif..** |
| Minimarket | 3.63% | 5.01% | 1.38% | 0.074 | . |
| Supermarket | 4.28% | 5.27% | 0.99% | 0.234 |  |
| Traditional market | 77.85% | 76.20% | -1.65% | 0.300 |  |
| Traveling merchant | 54.98% | 42.39% | -12.59% | <0.000 | *** |
| From fishers | 20.44% | 19.64% | -0.79% | 0.618 |  |
| Don’t consume/purchase | 1.52% | 2.70% | 1.19% | 0.032 | * |
| **Product type** | **Before** | **During** | **% change** | **P-value** | **Signif.** |
| Raw/fresh | 86.03% | 81.02% | -5.01% | <0.000 | *** |
| Cooked | 34.21% | 38.50% | 4.28% | 0.016 | * |
| Frozen | 5.34% | 6.39% | 1.05% | 0.247 |  |
| Canned | 4.22% | 4.09% | -0.13% | 0.928 |  |
| Dried/smoked | 19.58% | 21.89% | 2.31% | 0.128 |  |
| Don’t consume/purchase | 2.44% | 2.50% | 0.07% | 1.000 |  |
| Significance codes: <0.001 ‘***’ <0.01 ‘**’ <0.05 ‘*’ <0.1 ‘.’ | | | | | |

**Table S5.** Coefficients of the logistic regression models of the demographic and socioeconomic factors associated with seafood consumption change questions during COVID-19.

| Coefficients | **Estimate** | **Stand. error** | **t-value** | **Pr(>\|t\|)** | **Signif.** |
| --- | --- | --- | --- | --- | --- |
| **During COVID I ate less fisheries products than normal.** | | | | | |
| (Intercept) | 1.5072 | 0.4096 | 3.680 | 0.01430 | * |
| Eat fish 1-2 times per week | 0.7721 | 0.5478 | 1.410 | 0.21771 |  |
| Eat fish 3-4 times per week | -2.0586 | 0.4989 | -4.126 | 0.00912 | ** |
| Eat fish 5-6 times per week | -1.5773 | 0.5050 | -3.123 | 0.02615 | * |
| Eat fish 7+ times per week | -0.9689 | 0.4250 | -2.280 | 0.07158 | **.** |
| **During COVID, fisheries products were more expensive.** | | | | | |
| (Intercept) | 0.7349 | 0.1476 | 4.980 | 0.00108 | ** |
| Development: Rural | 0.2113 | 0.7102 | 0.297 | 0.77368 |  |
| **The fisheries products I usually buy or eat were not available during COVID.** | | | | | |
| (Intercept) | -1.7987 | 0.3143 | -5.723 | 0.00228 | ** |
| Gender: Male | 1.3197 | 0.5655 | 2.334 | 0.06689 | **.** |
| Island: Sumbawa | 1.7889 | 0.5935 | 3.014 | 0.02961 | * |
| Income: went down | 0.7368 | 0.5095 | 1.446 | 0.20778 |  |
| Income: went up | 2.9452 | 1.0911 | 2.699 | 0.04282 | * |
| **Fisheries products are an important part of food culture in the region.** | | | | | |
| (Intercept) | 4.4986 | 0.3176 | 14.163 | 0.00495 | ** |
| Development: Rural | -3.1631 | 0.8177 | -3.868 | 0.06080 | **.** |
| Age: 20-29 | 0.4655 | 1.0542 | 0.442 | 0.70194 |  |
| Age: 30-39 | 1.0007 | 1.5099 | 0.663 | 0.57567 |  |
| Age: 40-49 | -0.6504 | 0.1555 | -4.182 | 0.05270 | **.** |
| Age: 50-59 | 2.5265 | 1.3475 | 1.875 | 0.20163 |  |
| Age: 60+ | 19.2908 | 1.3674 | 14.107 | 0.00499 | ** |
| Gender: Male | -0.2978 | 0.3283 | -0.907 | 0.46004 |  |
| **I know the region where my fisheries products come from.** | | | | | |
| (Intercept) | 0.8783 | 0.7234 | 1.214 | 0.2915 |  |
| Age: 20-29 | -0.3558 | 0.7814 | -0.455 | 0.6725 |  |
| Age: 30-39 | 0.9006 | 1.1623 | 0.775 | 0.4817 |  |
| Age: 40-49 | 0.3148 | 0.7740 | 0.407 | 0.7051 |  |
| Age: 50-59 | 1.6423 | 0.9410 | 1.745 | 0.1559 |  |
| Age: 60+ | 4.4009 | 1.5585 | 2.824 | 0.0476 | * |
| **I prefer to buy fisheries products produced from West Nusa Tenggara.** | | | | | |
| (Intercept) | -0.28084 | 0.60594 | -0.463 | 0.654021 |  |
| Eat fish 1-2 times per week | 1.18149 | 0.62206 | 1.899 | 0.089986 | **.** |
| Eat fish 3-4 times per week | 2.97580 | 0.57288 | 5.194 | 0.000568 | *** |
| Eat fish 5-6 times per week | 2.16807 | 0.43799 | 4.950 | 0.000791 | *** |
| Eat fish 7+ times per week | 18.77537 | 0.83206 | 22.565 | 3.12e-09 | *** |
| Age: 20-29 | -0.05361 | 0.96442 | -0.056 | 0.956885 |  |
| Age: 30-39 | 3.28369 | 1.27954 | 2.566 | 0.030371 | * |
| Age: 40-49 | 1.47878 | 0.84130 | 1.758 | 0.112664 |  |
| Age: 50-59 | 2.47010 | 1.14848 | 2.151 | 0.059962 | **.** |
| Age: 60+ | 20.23082 | 1.44534 | 13.997 | 2.05e-07 | *** |
| **During COVID, I had enough money to buy the food I wanted.** | | | | | |
| (intercept) | -1.957083 | 0.249395 | -7.847 | 0.000226 | *** |
| Factor 2 - Income went down | 0.922884 | 0.924617 | 0.998 | 0.356755 |  |
| Factor 3 - Income went up | -0.009431 | 0.790651 | -0.012 | 0.990869 |  |
| Factor 1 - Male | 1.383428 | 0.426945 | 3.240 | 0.017680 | * |
| Significance codes: 0 ‘***’ 0.001 ‘**’ 0.01 ‘*’ 0.05 ‘.’ | | | | | |

**Table S6.** Full results for all COVID-19 Impact Index indicators contributing to aggregated index score.

| **Regency/Kota** | **Product Type Index** | **Buy Fresh Index** | **Buy Cooked Index** | **Could buy food Index** | **Ate less fish Index** | **Fish more expensive Index** | **Fish available Index** | **COVID Impact Index** |
| --- | --- | --- | --- | --- | --- | --- | --- | --- |
| **Kota Mataram** | 0.201 | 0.324 | 0.437 | 0.000 | 0.000 | 0.221 | 0.000 | 0.169 |
| **Lombok Barat** | 0.026 | 1.000 | 0.466 | 0.234 | 0.376 | 0.241 | 0.371 | 0.388 |
| **Lombok Utara** | 0.528 | 0.780 | 1.000 | 0.217 | 0.410 | 0.000 | 0.382 | 0.474 |
| **Lombok Tengah** | 0.000 | 0.838 | 0.658 | 0.955 | 0.613 | 0.608 | 0.698 | 0.624 |
| **Lombok Timur** | 0.457 | 0.551 | 0.319 | 1.000 | 0.558 | 0.549 | 0.513 | 0.564 |
| **Sumbawa Barat** | 1.000 | 0.739 | 0.642 | 0.618 | 0.785 | 1.000 | 0.855 | 0.806 |
| **Sumbawa** | 0.102 | 0.260 | 0.428 | 0.908 | 0.585 | 0.539 | 0.915 | 0.534 |
| **Dompu** | 0.904 | 0.597 | 0.000 | 0.367 | 0.341 | 0.542 | 0.743 | 0.499 |
| **Bima** | 0.472 | 0.630 | 0.197 | 0.476 | 1.000 | 0.587 | 1.000 | 0.623 |
| **Kota Bima** | 0.682 | 0.000 | 0.463 | 0.582 | 0.300 | 0.447 | 0.530 | 0.429 |

**Table S7.** Gender income averages before and during COVID-19.

| **Gender** | **Income classification average before** | **Income classification average during** | **Mean difference before and during** | **Mann-Whitney U test** |
| --- | --- | --- | --- | --- |
| Men | mean = 1.885, median = 2, n = 696 | mean = 1.615, median = 1, n = 696 | 0.27 | p-value = 2.152e-08, z = 9.8 |
| Women | mean = 1.629, median = 1, n = 821 | mean = 1.479, median = 1, n = 821 | 0.15 | p-value = 9.228e-06, z = 7.6 |
| Difference between gender income means | 0.256 | 0.136 |  |  |

**Table S8.** Education level and the average number of seafood species consumed per year.

| **Education level** | **Average number of seafood species consumed per year** |
| --- | --- |
| 1 | 2.12 |
| 2 | 3.04 |
| 3 | 4.62 |
| 4 | 6.43 |

**Table S9.** Location and the average number of seafood species consumed per year.

| **Regency** | **Average number of seafood species consumed per year** |
| --- | --- |
| **Kota Mataram** | 6.74 |
| **Lombok Barat** | 6.28 |
| **Lombok Utara** | 5.8 |
| **Lombok Tengah** | 4.35 |
| **Lombok Timur** | 4.70 |
| **Sumbawa Barat** | 4.69 |
| **Sumbawa** | 5.36 |
| **Dompu** | 6.33 |
| **Bima** | 4.14 |
| **Kota Bima** | 5.59 |

**One-way Anova: Mean Seafood Species Consumed x Regency/Kota**


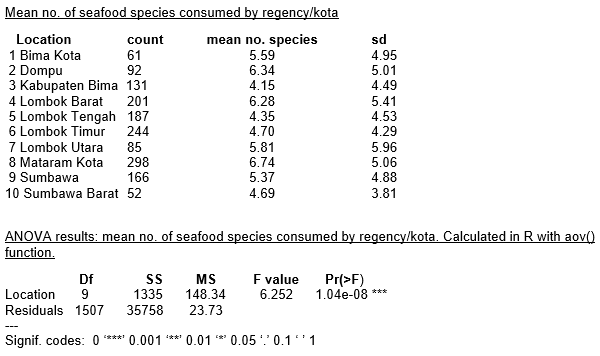


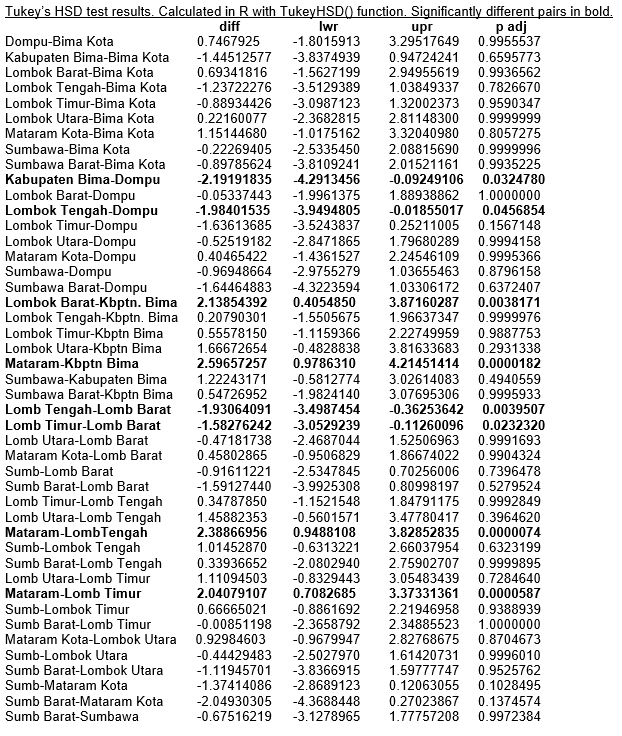


**One-way Anova: Mean Seafood Species Consumed x Income(before COVID)**


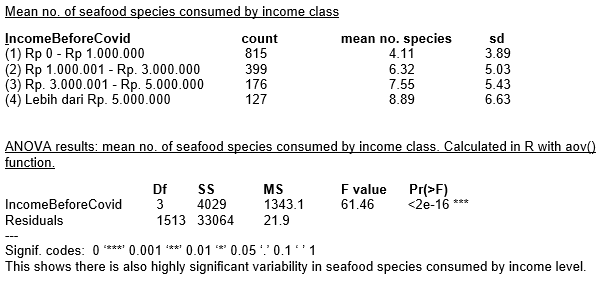


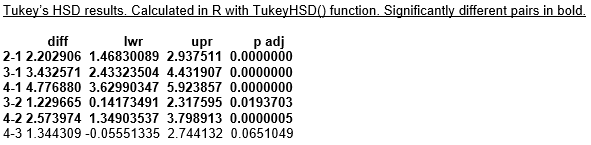


**One-way Anova: Mean Seafood Species Consumed x Education**


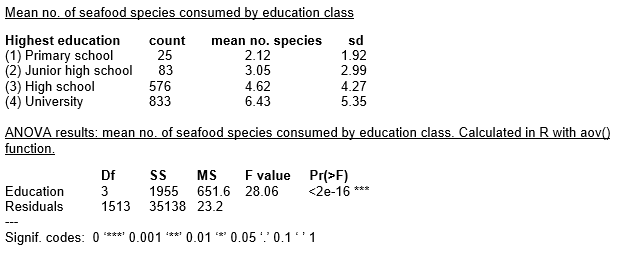


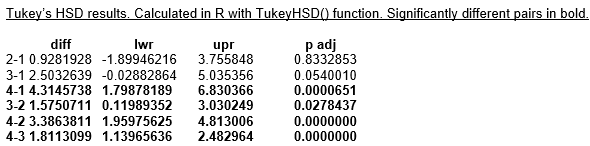


**Section 2: COVID seafood consumption impact index**

**Seafood survey: COVID impact index**

In order to compare patterns of COVID-19 impacts across regions of Nusa Tenggara Barat, a simple impact index was developed from seven survey questions which particularly pertained to changes in seafood consumption as a result of the pandemic. This data was used to calculate an aggregated COVID impact index, with scores ranging from 0 (minimum impact) to 1 (maximum impact), which is calculated per regency/kota from the average of 7 impact indicators. Raw survey data as well as columns for each indicator and district can be found in “SeafoodIndex.csv”. R code can be found in “COVIDIndexRscript.R”. A short description of each of the seven indicators and how they were calculated follows below.

**Indicator 1: Ate less fish due to COVID “AtelessfishIndex”**

Survey question (English): During the COVID-19 pandemic, I ate less seafood than usual.

Survey question (Bahasa): Selama pandemi COVID-19, saya makan hasil laut dan budidaya perikanan berkurang dari biasanya

|  | Raw Survey score | Transformed index calculation score |
| --- | --- | --- |
| Agree | 1 | 1 |
| Disagree | 2 | 0 |
| Don’t eat fish | 3 | na |

This score was calculated as the proportion of total respondents per region who answered “agree”.

**Indicator 2: FishmoreexpensiveIndex**

Survey question (English): During the Covid-19 pandemic, the price of seafood became more expensive.

Survey question (Bahasa): Selama pandemi Covid-19, harga produk hasil laut dan budidaya perikanan menjadi lebih mahal.

|  | Raw Survey score | Transformed index calculation score |
| --- | --- | --- |
| Agree | 1 | 1 |
| Disagree | 2 | 0 |
| Don’t eat fish | 3 | na |

This score was calculated as the proportion of total respondents per region who answered “agree”.

**Indicator 3: FishavailableIndex**

Survey question (English): During the Covid-19 pandemic, the seafood products I used to eat or buy became unavailable.

Survey question (Bahasa): Selama pandemi Covid-19, saya makan hasil laut dan budidaya perikanan berkurang dari biasanya

|  | Raw Survey score | Transformed index calculation score |
| --- | --- | --- |
| Agree | 1 | 1 |
| Disagree | 2 | 0 |
| Don’t eat fish | 3 | na |

This score was calculated as the proportion of total respondents per region who answered “agree”.

**Indicator 4: CouldbuyfoodIndex**

Survey question (English): During the Covid-19 pandemic, I had enough money to buy the food I wanted.

Survey question (Bahasa): Selama pandemi Covid-19, saya memiliki cukup uang untuk membeli makanan yang saya inginkan

|  | Raw Survey score | Transformed index calculation score |
| --- | --- | --- |
| Agree | 1 | 0 |
| Disagree | 2 | 1 |

As this question had a positive framing, in this score was calculated as the proportion of total respondents per district who answered “disagree”.

**Indicator 5: ProductTypeIndex**

Survey question (English): Before the Covid-19 pandemic, what seafood products were you buying or consuming? + During the Covid-19 pandemic, what seafood products are you buying or consuming?

Survey question (Bahasa): Sebelum pandemi Covid-19, produk hasil laut dan budidaya perikanan apa saja yang biasa Anda beli atau konsumsi? + Selama pandemi Covid-19, produk hasil laut dan budidaya perikanan apa saja yang biasa Anda beli atau konsumsi?

This indicator is an average measure of change in seafood product type consumption during the pandemic. The columns listed below in the dataset each represent the *absolute* difference in the binary yes/no scores between the before and during columns of each product type. Therefore, a 1 represents a change in product type habits for each product type before and during COVID. Dropping a product type and adding a product type before/during COVID are therefore treated equally, in essence a measure of overall change before and during the pandemic. The final indicator averaging across product types is the “ProductTypeIndex” column, one of seven indicators in the final index.

| ProductTypeRaw | ProductTypeCooked | ProductTypeFrozen | ProductTypeCanned | ProductTypeDried |
| --- | --- | --- | --- | --- |

**Indicator 6: BuyFreshIndex**

Survey question (English): Before the Covid-19 pandemic, where did you usually buy seafood products that were raw/fresh? + During the Covid-19 pandemic, where do you usually buy seafood products that are raw/fresh?

Survey question (Bahasa): Sebelum pandemi Covid-19, dimana Anda biasa membeli produk hasil laut dan budidaya perikanan yang masih mentah? + Selama pandemi Covid-19, dimana Anda biasa membeli produk hasil laut dan budidaya perikanan?

This indicator is an average measure of change in fresh seafood purchasing location during the pandemic. The columns listed below in the dataset each represent the *absolute* difference in the binary yes/no scores between the before and during columns of each purchasing location. Therefore, a score of 1 represents a change in purchasing location before and during COVID. Dropping a purchasing location and adding a purchasing location before/during COVID are therefore treated equally, in essence a measure of overall change before and during the pandemic. The final indicator averaging across purchasing locations is the “BuyFreshIndex” column, one of seven indicators in the final index.

| BuyFreshMiniMkt | BuyFreshSuperMkt | BuyFreshTrdtnlMrkt | BuyFreshTravMerch | BuyFreshFishermen |
| --- | --- | --- | --- | --- |

**Indicator 7: BuyCookedIndex**

Survey question (English): Before the Covid-19 pandemic, where did you usually buy cooked seafood products? + During the Covid-19 pandemic, where do you usually buy cooked seafood products?

Survey question (Bahasa): Sebelum pandemi Covid 19, dimana Anda biasa membeli produk hasil laut dan budidaya perikanan yang sudah dimasak? + Selama pandemi Covid 19, dimana Anda biasa membeli produk hasil laut dan budidaya perikanan yang sudah dimasak?

This indicator is an average measure of change in cooked seafood purchasing location during the pandemic. The columns listed below in the dataset each represent the *absolute* difference in the binary yes/no scores between the before and during columns of each purchasing location. Therefore, a score of 1 represents a change in purchasing location before and during COVID. Dropping a purchasing location and adding a purchasing location before/during COVID are therefore treated equally, in essence a measure of overall change before and during the pandemic. The final indicator averaging across purchasing locations is the “BuyCookedIndex” column, one of seven indicators in the final index.

| BuyCookedMiniMkt | BuyCookedSuperMkt | BuyCookedTrdtnlMrkt | BuyCookedStreetV | BuyCookedShop | BuyCookedRrstnt |
| --- | --- | --- | --- | --- | --- |

**Score calculations and final aggregated COVID impact index**

To calculate the overall COVID impact index, the average score for each of the seven indicators was calculated for each of the 10 regency/kota in Nusa Tenggara Barat. Each indicator was then normalized from 0-1, representing the degree of relative impact per indicator across regions. All seven indicators then contributed equally to the aggregated COVID impact index score, which averaged the seven indicator scores for each region.

**Section 3: Survey**

**Bahasa-Indonesia original**

| **Survey berdurasi 5 menit terkait produk-produk perikanan dan Covid-19 di Lombok** | | |
| --- | --- | --- |
| **Informasi Dasar** | | |
| Setiap orang yang tinggal di Nusa Tenggara Barat dapat melengkapi survey ini. Setiap responden mendapatkan Rp. 10.000 pulsa dan hanya boleh berpartisipasi **satu kali.** Survey dilaksanakan selama 6 minggu dan pulsa telepon akan diberikan setelah periode survey berakhir. Informasi dan data yang didapatkan dari survey ini akan dijaga kerahasiaannya dan digunakan untuk riset.  Riset ini dilakukan oleh Leibniz Centre for Tropical Marine Research (ZMT), Jerman. <https://www.leibniz-zmt.de/en/>  Setuju dan Lanjutkan □ | | |
| 1 | Nomor telepon (untuk mendapatkan pulsa) | [fill in] |
| 2 | Usia | Dibawah 20  20-29  30-39  40-49  50-59  60+ |
| 3 | Jenis kelamin | Perempuan  Laki laki |
| 4a | Kabupaten | Mataram Kota  Lombok Barat  Lombok Utara  Lombok Tengah  Lombok Timur  Sumbawa Barat  Sumbawa  Dompu  Kabupaten Bima  Bima Kota |
| 4b | Kecamatan | Kecamatan list |
| 5 | Jumlah anggota keluarga yang tinggal bersama di rumah Anda | 0 1 2 3 4 5+ |
| 6 | Pendidikan terakhir | Sekolah dasar  Sekolah menengah pertama  Sekolah menengah atas/ kejuruan  Universitas |
| 7 | Pekerjaan | Bekerja penuh waktu  Bekerja paruh waktu  Pelajar  Wirausaha / memiliki bisnis  Ibu rumah tangga  Tidak bekerja |
| 8 | Pendapatan perbulan | Rp 0 - Rp 1.000.000  Rp 1.000.001 - Rp. 3.000.000  Rp. 3.000.001 - Rp. 5.000.000  Lebih dari Rp. 5.000.000 |
|  | Pengeluaran perbulan | Rp 0 - Rp 1.000.000  Rp 1.000.001 - Rp. 3.000.000  Rp. 3.000.001 - Rp. 5.000.000  Lebih dari Rp. 5.000.000 |
|  | **Perilaku konsumsi produk perikanan** | |
| Pertanyaan berikut ini berkaitan dengan perilaku konsumsi produk-produk perikanan sebelum dan selama pandemi Covid-19. Produk perikanan yang dimaksud dalam konteks ini adalah semua yang berasal dari air tawar, air payau, dan air laut seperti ikan bandeng, selar, tuna/tongkol/cakalang, sarden, kerapu, nila/mujair, teri, lele, mas, patin, belt, gabu, bawal, kembung, kakap putih, tenggiri, pari, ekor kuning lobster, kepiting, udang, tiram, kerang-kerangan, cumi-cumi, gurita, sotong, produk olahan rumput laut, dan ikan asin, dan lainnya. | | |
| 9 | Seberapa sering anda mengkonsumsi produk-produk perikanan per minggu? | 0  1-2 kali  3-4 kali  5-7 kali  8+ |
| 10 | Selama pandemi Covid-19,konsumsi produk-produk perikanan saya berkurang dari biasanya | Setuju  Tidak Setuju  Saya tidak memakan atau membeli produk perikanan |
| 11 | Selama pandemi Covid-19, harga produk-produk perikanan menjadi lebih mahal | Setuju  Tidak Setuju  Saya tidak memakan atau membeli produk perikanan |
| 12 | Selama pandemi Covid-19,konsumsi produk-produk perikanan yang biasa saya makan atau beli menjadi tidak tersedia | Setuju  Tidak Setuju  Saya tidak memakan atau membeli produk perikanan |
| 13 | Produk-produk perikanan adalah bagian penting dalam budaya di Lombok. | Setuju  Tidak Setuju |
|  | Saya mengetahui daerah dari mana produk-produk perikanan saya berasal | Setuju  Tidak Setuju  Saya tidak memakan atau membeli produk perikanan |
| 14 | Saya lebih memilih produk-produk perikanan yang berasal dari Nusa Tenggara Barat. | Setuju  Tidak Setuju  Saya tidak memakan atau membeli produk perikanan |
| 15 | Selama pandemi Covid-19, saya memiliki cukup uang untuk membeli makanan yang saya inginkan | Setuju  Tidak Setuju |
| 16 | Protein apa saja yang Anda konsumsi paling tidak sekali dalam seminggu (pilih semua yang sesuai) | Check boxes:  Daging  Seafood  Ayam  Domba / Kambing  Kedelai / Tempe / Tahu |
| 17 | Jenis ikan apa saja yang Anda konsumsi? (pilih semua yang sesuai) | Check boxes:  Ikan bandeng  Ikan selar  Ikan tuna, tongkol, cakalang  Ikan sarden  Ikan salmon  Ikan kerapu  Ikan nila  Ikan mujair  Ikan teri  Ikan lele  Ikan mas  Ikan patin  Belut  Ikan gabus  Ikan bawal  Ikan kembung  Ikan kakap putih  Ikan tenggiri  Ikan pari  Ikan ekor kuning  Lobster  Kepiting  Udang  Tiram  Kerang-kerangan  Cumi-cumi  Gurita  Sotong  Produk olahan rumput laut  Ikan asin/kering  Saya tidak memakan atau membeli seafood  Lainnya, sebutkan_____ |
| 18 | Sebelum pandemi Covid-19, produk-produk perikanan apa saja yang biasa Anda beli atau konsumsi? | Check boxes:  Mentah dan segar  Sudah dimasak  Beku  Kalengan  Kering/asap  Saya tidak memakan atau membeli seafood |
| 19 | Selama pandemi Covid-19, produk-produk perikanan apa saja yang biasa Anda beli atau konsumsi? | Check boxes:  Mentah dan segar  Sudah dimasak  Beku  Kalengan  Kering/asap  Saya tidak memakan atau membeli seafood |
| 20 | Sebelum pandemi Covid-19, dimana Anda biasa membeli produk-produk perikanan mentah yang segar atau beku? | Check boxes:  Minimarket (seperti Alfa, Indomaret, dsb)  Supermarket (seperti Giant, Carrefour, dsb)  Pasar tradisional  Pedagang keliling  Nelayan atau pembudidaya perikanan  Saya tidak memakan atau membeli seafood  Lainnya, sebutkan…... |
| 21 | Selama pandemi Covid-19, dimana Anda biasa membeli produk-produk perikanan mentah yang segar atau beku? | Check boxes:  Minimarket (seperti Alfa, Indomaret, dsb)  Supermarket (seperti Giant, Carrefour, dsb)  Pasar tradisional  Pedagang kaki lima  Nelayan atau pembudidaya perikanan  Saya tidak memakan atau membeli seafood  Lainnya, sebutkan…... |
| 22 | Sebelum pandemi Covid 19, dimana Anda biasa membeli produk-produk perikanan yang sudah dimasak? | Check boxes:  Minimarket (seperti Alfa, Indomaret, dsb)  Supermarket (seperti Giant, Carrefour, dsb)  Pasar tradisional  Pedagang kaki lima  Warung  Restoran  Saya tidak memakan atau membeli seafood  Lainnya, sebutkan…... |
| 23 | Selama pandemi Covid 19, dimana Anda biasa membeli produk-produk perikananyang sudah dimasak? | Check boxes:  Minimarket (seperti Alfa, Indomaret, dsb)  Supermarket (seperti Giant, Carrefour, dsb)  Pasar tradisional  Warung  Restoran  Saya tidak memakan atau membeli seafood  Lainnya, sebutkan…. |
| 24 | Komen tambahan jika diperlukan | [Fill in] |

**English original**

| **Basic information** | | |
| --- | --- | --- |
| Everyone living in West Nusa Tenggara can complete this survey. Each respondent gets Rp. 10,000 credits and can only participate once. The survey is carried out for 6 weeks and telephone credit will be given after the survey period ends. Information and data obtained from this survey will be kept confidential and used for research.  This research was conducted by the Leibniz Center for Tropical Marine Research (ZMT), Germany.  <https://www.leibniz-zmt.de/en/>  Agree and continue □ | | |
| 1 | Phone number (to receive phone credit) | [fill in] |
| 2 | Age | Under 20  20-29  30-39  40-49  50-59  60+ |
| 3 | Gender | Female Male |
| 4a | Kabupaten | Mataram City  West Lombok  North Lombok  Central Lombok  East Lombok  West Sumbawa  Sumbawa  Dompu  Bima District  Bima City |
| 4b | Kecamatan | Kecamatan list |
| 5 | Number of people or family members living together in your house | 0 1 2 3 4 5+ |
| 6 | Completed education level | Elementary school  Junior high school  High school  University or higher |
| 7 | Occupation | Working full time  Working part time  Student  Entrepreneur / own business  Housewife  Unemployed |
| 8 | Individual income per month | Rp 0 - Rp 1.000.000  Rp 1.000.001 - Rp. 3.000.000  Rp. 3.000.001 - Rp. 5.000.000  More than Rp. 5.000.000 |
|  | Individual expenditure per month | Rp 0 - Rp 1.000.000  Rp 1.000.001 - Rp. 3.000.000  Rp. 3.000.001 - Rp. 5.000.000  More than Rp. 5.000.000 |
|  | **Fisheries consumption behavior** | |
| The following questions are related to your food consumption behaviour before and during Covid-19 pandemic. The definition fisheries products sourced from fresh, brackish, and salt water resource systems such as milkfish, mackerel, tuna, sardine, salmon, grouper, tilapia, anchovy, catfish, carps, pangasius, lobster, crab, shrimp, oyster, shellfish, squid, octopus, cuttlefish, seaweed products, and dried/ salted fish etc. | | |
| 9 | How often do you eat fisheries products  per week on average? | 0  1-2 times  3-4 times  5-7 times  8+ |
| 10 | During COVID I ate less fisheries products than normal. | Agree  Disagree  I don't eat or buy seafood |
| 11 | During COVID, fisheries products were more expensive. | Agree  Disagree  I don't eat or purchase seafood |
| 12 | The fisheries products I usually buy or eat were not available during COVID. | Agree  Disagree  I don't eat or buy seafood |
| 13 | The fisheries products are an important part of food culture in Lombok. | Agree  Disagree |
|  | I know the region where my fisheries products come from. | Agree  Disagree  I don't eat or buy seafood |
| 14 | I prefer to buy fisheries products produced from West Nusa Tenggara. | Agree  Disagree  I don't eat or buy seafood |
| 15 | During COVID, I had enough money to buy the food I wanted. | Agree  Disagree |
| 16 | What kind of protein do eat at least one time per week (check all that apply) | Check boxes:  Beef  Seafood  Lamb  Chicken  Soya/Tempe/Tahu |
| 17 | What kind of fisheries products do you eat normally throughout the year? (check all that apply) | Check boxes:  Milkfish  Mackerel  Tuna  Sardine  Salmon  Grouper  Nile Tilapia  Tilapia  Anchovy  Catfish  Carps  Pangasius  Eel  Snakehead fish / murrel  Pomfret fish  Long jawed mackerel  Seabass  Spanish mackerel fish  Stingray / rayfish  Yellow tail fish  Lobster  Crab  Shrimp  Oyster  Shellfish  Squid  Octopus  Cuttlefish  Processed seaweed products  Salted and dried fish  Other______________  I don't eat or buy seafood |
| 18 | Before Covid 19, I usually buy/eat fisheries products the following type: | Check boxes:  Raw and fresh  Cooked  Frozen  Canned  Dried/smoked  I don't eat or buy seafood |
| 19 | During Covid 19, I usually buy/eat fisheries products the following type:: | Check boxes:  Raw and fresh  Cooked  Frozen  Canned  Dried/smoked  I don't eat or buy seafood |
| 20 | Before Covid 19, where do you usually buy fresh or frozen fisheries products?: | Check boxes:  Minimarket (e.g., Alfa, Indomaret)  Supermarket (e.g., Giant, Carrefour)  Traditional markets  Street vendors  Fishermen  I don't eat or buy seafood  Other……. |
| 21 | During Covid 19, where do you usually buy fresh or frozen fisheries products? | Check boxes:  Minimarket (e.g., Alfa, Indomaret)  Supermarket (e.g., Giant, Carrefour)  Traditional markets  Street vendors  Fishermen  I don't eat or buy seafood  Other……. |
| 22 | Before Covid 19, where do you usually buy cooked fisheries products? | Check boxes:  Minimarket (e.g., Alfa, Indomaret)  Supermarket (e.g., Giant, Carrefour)  Traditional markets  Street vendors  Warung  Restaurants  I don't eat or buy seafood  Other……. |
| 23 | During Covid 19, where do you usually buy fresh or frozen fisheries products: | Check boxes:  Minimarket (e.g., Alfa, Indomaret)  Supermarket (e.g., Giant, Carrefour)  Traditional markets  Street vendors  Warung  Restaurants  I don't eat or buy seafood  Other……. |
| 24 | Additional comments | [Fill in] |
