# Supplementary material for: Seafood consumption changes and COVID-19 impact index in West Nusa Tenggara, Indonesia
Source: PLoS One. 2023 Jan 18;18(1):e0280134. doi: 10.1371/journal.pone.0280134 (PMC9847987; doi:10.1371/journal.pone.0280134)
Supplement: S3 File — (DOCX) [file pone.0280134.s003.docx]

**Abstrak (Bahasa-Indonesia)**

Penelitian ini mengkaji pola perubahan konsumsi produk perikanan di tingkat kabupaten di Nusa Tenggara Barat, Indonesia, selama wabah virus COVID-19 (SARS-Cov-2) hingga 31 Desember 2020. Metode penelitian menggunakan stratified semi-random survey yang dikelola secara online melalui Whatsapp dan Facebook untuk penilaian dan penyebaran cepat dengan total sampel 1518 responden. Berdasarkan hasil survei, kami membuat indeks dampak COVID-19 pada tingkat kabupaten yang mengindikasikan pola perubahan konsumsi produk perikanan dalam bentuk gradien di daerah perkotaan hingga pedesaan, dengan hasil daerah pedesaan banyak mengalami perubahan. Selama COVID-19, 61% responden mengkonsumsi lebih sedikit seafood dari normal, 66% setuju bahwa produk perikanan menjadi lebih mahal, dan 37% setuju produk perikanan yang biasa dibeli menjadi tidak tersedia. Pada perbandingan konsumsi sebelum dan selama COVID-19, 5% respondent membeli lebih sedikit produk perikanan mentah dan segar; 4,3% membeli 4.3% produk olahan ikan. Pasar tradisional, pedagang keliling, dan warung makan tetap menjadi tujuan utama konsumen untuk membeli produk perikanan, meskipun terjadi penurunan akses selama pandemi di semua gerai, mini dan supermarket hanya mengalami sedikit penurunan. Pembelian produk perikanan mentah dan segar melalui pedagang keliling menurun 12,5% selama pandemi. Terdapat presentasi yang lebih besar untuk perempuan yang mengkonsumsi produk perikanan sekali dalam seminggu (~10% lebih daripada laki laki) dengan produk perikanan yang lebih beragam. Secara keseluruhan, responden yang mengindikasikan memakan produk perikanan lebih sering dalam seminggu, adalah responden yang lebih cenderung setuju untuk memakan lebih sedikit ikan selama pandemi. Responden dari Pulau Sumbawa cenderung setuju jika produk perikanan menjadi tidak tersedia selama pandemi.
